# The 2‑Amino-3,4-dihydroquinazoline Molecular Scaffold as Novel OCT3 Inhibitor

**DOI:** 10.1021/acschemneuro.5c01026

**Published:** 2026-03-20

**Authors:** Kavita A. Iyer, Xiaolei Pan, Charles B. Jones, Hebing Liu, Malaika D. Argade, Osama I. Alwassil, Douglas H. Sweet, Małgorzata Dukat

**Affiliations:** † Department of Medicinal Chemistry, School of Pharmacy, Virginia Commonwealth University, Richmond, Virginia 23298, United States; ‡ Department of Pharmaceutics, School of Pharmacy, 4137 Virginia Commonwealth University, Richmond, Virginia 23298, United States

**Keywords:** SLC22 transporter family, SLC22A1−3, OCT inhibition, small molecules, 3D molecular modeling, mouse-tail suspension test

## Abstract

Organic cation transporters 1–3 (OCTs 1–3), especially OCT3, have emerged as “high-capacity” uptake transporters for the aminergic neurotransmitters serotonin, norepinephrine, and dopamine from the synapse. We previously reported the 6- and 7-chloro analogs of 2-aminodihydroquinazoline (i.e., A6CDQ and A7CDQ, respectively) as novel inhibitors of OCTs. Here, we synthesized and evaluated a focused series of analogs bearing substituents at the aryl 5-, 6-, 7-, or 8-position. All compounds inhibited action at OCT1, OCT2, and especially OCT3. The present study centered primarily on OCT3 because it has been implicated in the action of antidepressants. Through this work, seven analogs were found to be more potent, or at least equipotent, at OCT3 than A6CDQ or A7CDQ. Additionally, three analogs were found, as with A6CDQ and A7CDQ, to be active in the mouse tail suspension test – a well-established proxy for evaluating potential antidepressant-like action. Our 3D molecular modeling studies identified SER474, ASP478, and CYS477 as key residues in the binding interactions of the 2-aminodihydroquinazoline (ADQ) chemotype at OCT3. Furthermore, the binding mode of ADQ analogs and the extensive size of the binding pocket warrant further examination of the scaffold, and particularly of additional aryl substituents to exploit this region of bulk tolerance.

## Graphical abstract

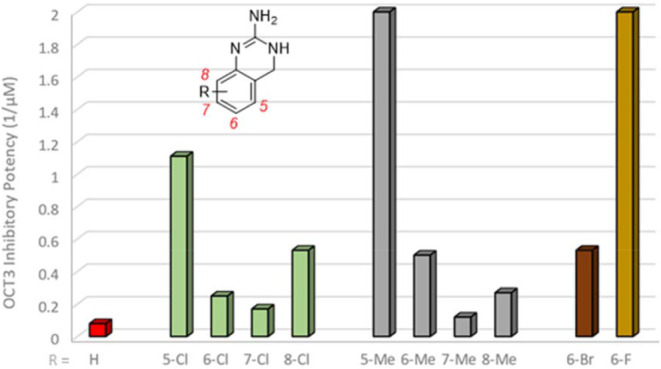

## Introduction

At least three dozen agents are clinically available for the treatment of depression, and most act by enhancing the neurotransmission of serotonin or norepinephrine, or both, by blocking their reuptake, or by interfering with their metabolism. Some newer antidepressants directly target serotonin receptors. Nevertheless, despite their general utility, onset of action is usually delayed, >30% of patients fail to respond to treatment, and various side effects (e.g., adverse cardiac events, anticholinergic action, hypotension, sedation) are not uncommon due to their off-target actions (reviewed ref 1). Attention has recently refocused on developing agents with novel (or multimodal) mechanisms of action.^1^

Various psychotherapeutic agents act via monoamine transporters (MATs) (i.e., serotonin, norepinephrine, and dopamine transporters, SERT, NET, and DAT, respectively), members of the SLC6 transporter family. However, there are at least two distinguishable mechanisms of aminergic neurotransmitter clearance in the synapse: uptake-1 (high-affinity, low-capacity transporters; i.e., MATs) and the underexplored uptake-2 (low-affinity, high-capacity organic cation transporters: hOCT1–3, members of the SLC22 family). Although OCTs are expressed and located in various human organs and tissues, all three are significantly expressed in human brain and brain microvessels with OCT2 and OCT3 located in neurons and only OCT3 located both in neurons and glial cells (reviewed ref 2). Furthermore, OCT3 is the most highly expressed OCT in the human blood-brain barrier.^3^ Xenobiotics from a variety of therapeutic classes (e.g., antidepressants, anxiolytics, sedatives) interact with OCTs as low-affinity substrates or inhibitors even though their primary mechanism of action is associated with much higher affinity at some other transporter or receptor.^2,4^ A number of known antidepressants, such as desipramine, imipramine, amitriptyline, sertraline and fluoxetine (FLX), were shown to inhibit OCT3 and, thus, increase synaptic concentrations of biogenic amines.^5^ Also, the antidepressant-like effect of fluvoxamine was shown to be enhanced in the presence of decynium-22 (D-22), a known inhibitor of OCTs,^6–9^ in wild-type mice in the mouse tail suspension test (TST), a rodent model used for the evaluation of novel antidepressants.^10^ This effect was shown to be decreased in OCT3 knockout mice.^11^ Therefore, OCT3 presents an attractive target for the development of a mechanistically novel class of antidepressant agents. Further studies are warranted by the lack of specific inhibitors and the availability of the recently reported cryo-EM structure of hOCT3.^12^

Previously, we introduced phenylguanidines as a novel structural scaffold for agents that act as inhibitors at OCTs.^13^ A conformationally constrained phenylguanidine analog, 2-amino-6-chloro-3,4-dihydroquinazoline (A6CDQ; **1**), was initially developed by us as a 5-HT_3_ receptor antagonist.^14^ A6CDQ (**1**) and one of its positional isomers, the 7-chloro analog A7CDQ (**2**), produced antidepressant-like effects in the mouse TST. Activities at the 5-HT_3_ receptors or the serotonin transporter (SERT) were considered as potential mechanisms for this effect. However, we investigated and systematically eliminated a solely 5-HT_3_ receptor- or hSERT-mediated mechanism of antidepressant-like effects for both A6CDQ and A7CDQ, but found that both quinazolines inhibit OCT3.^15^

A major goal of the present study was to investigate the as yet unexplored structure–activity relationships (SARs) of 2-aminodihydroquinazoline (ADQ) analogs at OCT3 as compared to **1** and **2**. Thus, we examined (i) the role (and position) of the chloro group, (ii) the electronic/lipophilic effects of substituents at the 6-position, and (iii) the molecular interactions between ADQ analogs and a hOCT3 3D model. Selected ADQ analogs were further evaluated for antidepressant-like potential in the mouse TST.

## Results

### Synthesis

Compounds **1**, **2**,^14^ **3**,^16^ **4**,^17^ the chloro-substituted ADQ positional isomers, and their unsubstituted parent compound **5**,^16^ were synthesized as their hydrochloride salts (see Table 1 for structures) as previously reported. The remaining compounds were synthesized as shown in Scheme 1.

**Scheme 1:**
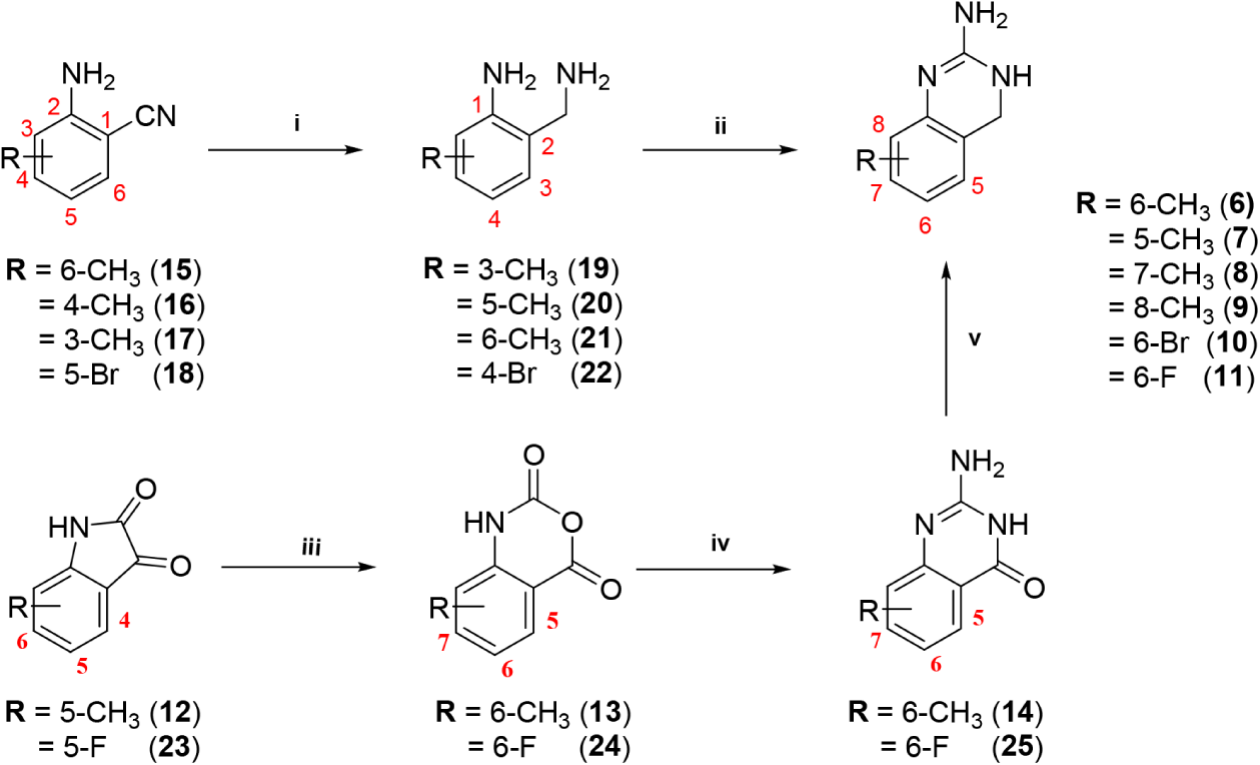
Synthesis of Target Compounds **6**–**11** ^a^

a Reagents and conditions: (i) BH_3_-THF, Δ, 12 h, 6 N HCl, 3 N NaOH, CH_2_Cl_2_; (ii) BrCN, PhMe, Δ, 12 h; (iii) H_2_O_2_, CH_3_COOH/H_2_SO_4_, sonication; (iv) CH_3_SC­(NH)­NH_2_·H_2_SO_4_, Na_2_CO_3_, 80% aq. MeCN, Δ, 6h; (v) BH_3_-THF, Δ, 2.5 h, 3 N NaOH, CHCl_3_.

**Table 1:**
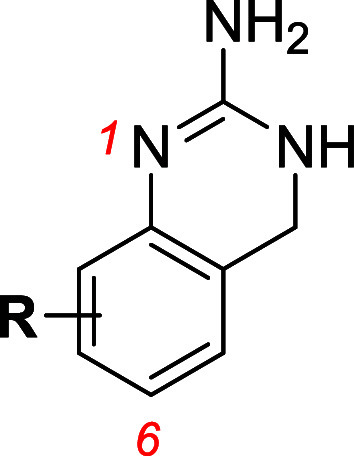
Inhibitory Potencies (IC_50_ ± SEM μM) of ADQ Analogs at hOCT1–3

Analog	R	hOCT1	hOCT2 IC_50_ ± SEM (μM)	hOCT3
A6CDQ (**1**)	6-Cl	3.0^a^	16.4^a^	3.9^a^
A7CDQ (**2**)	7-Cl	4.8^a^	9.2^a^	5.9^a^
A5CDQ (**3**)	5-Cl	2.3 ± 0.5	9.2 ± 0.8	0.9 ± 0.1
A8CDQ (**4**)	8-Cl	6.1 ± 0.2	26.1 ± 11.4	1.9 ± 0.1
ADQ (**5**)	H	14.3 ± 2.2	46.5 ± 0.6	12.2 ± 1.9
A6MDQ (**6**)	6-CH_3_	8.2 ± 2.2	12.1 ± 0.3	2.0 ± 0.4
A5MDQ (**7**)	5-CH_3_	4.6 ± 0.6	26.4 ± 0.7	0.5 ± 0.0
A7MDQ (**8**)	7-CH_3_	6.3 ± 0.7	12.1 ± 1.3	8.4 ± 2.5
A8MDQ (**9**)	8-CH_3_	4.5 ± 0.9	35.1 ± 6.7	3.7 ± 0.2
A6BrDQ (**10**)	6-Br	2.7 ± 0.4	9.3 ± 0.6	1.9 ± 0.9
A6FDQ (**11**)	6-F	1.3 ± 0.1	11.9 ± 0.2	0.5 ± 0.0

a Data previously reported by us.^15^

A6MDQ (**6**) was synthesized in a concise 3-step route starting with the oxidation of 5-methylisatin (**12**) with a urea-H_2_O_2_ complex under acidic conditions using ultrasonic irradiation to give 6-methylisatoic anhydride (**13**). The anhydride **13** was converted to 2-amino-6-methylquinazolin-4­(3*H*)-one (**14**) by condensation with S-methylpseudothiourea sulfate. Subsequent reduction of the amide group of **14** using BH_3_·THF complex as a hydride source yielded A6MDQ (**6**). The synthesis of 2-amino-5-methyl-3,4-dihydroquinazoline (A5MDQ; **7**), 2-amino-7-methyl-3,4-dihydroquinazoline (A7MDQ; **8**) and 2-amino-8-methyl-3,4-dihydroquinazoline (A8MDQ; **9**) involved cyclization of the appropriately substituted 2-aminobenzylamines (**19**–**22**) with cyanogen bromide as shown in Scheme 1. The intermediate benzylamines **19**–**22** were obtained by reduction of a nitrile group on appropriately substituted 2-aminobenzonitriles (**15**–**18**) with BH_3_·THF complex. In order to further probe the 6-position with respect to steric properties and bulk, we synthesized additional halo-substituted 2-aminodihydroquinazoline analogs (i.e., 6-F and 6-Br). For A6FDQ (**11**), the known corresponding 6-fluoroisatoic anhydride (**24**) was prepared according to a literature procedure^18^ and converted to 2-amino-6-fluoroquinazolin-4­(*3H*)-one (**25**) followed by reduction of amide group to give A6FDQ (**11**). 2-Amino-6-bromo-3,4-dihydroquinazoline (A6BrDQ; **10**) was synthesized following the route for preparation of **7**–**9** (Scheme 1). All target structures were prepared as water-soluble hydrochloride/hydrobromide salts except for free base **7** and confirmed by IR, ^1^H NMR, and elemental analysis for C, H, N.

### hOCT1, hOCT2, and hOCT3 Inhibition

Inhibition of hOCT-mediated MPP^+^ (1-methyl-4-phenylpyridinium) transport activity, and lack of significant background accumulation in empty vector control cells, was confirmed at a fixed concentration of 500 μM for each test compound on each transporter prior to kinetic analysis and IC_50_ calculation (data not shown). Each of the compounds exhibited significant (>50%) inhibition of hOCT1–3 transport under this condition; thus, all compounds were further examined. For kinetic analysis, [^3^H]­MPP^+^ (1 μM) accumulation in HEK293 cells stably expressing hOCT1, hOCT2 or hOCT3 was determined in the absence or presence of increasing concentrations (1 × 10^–8^ to 1 × 10^–3^ M) of the eleven 2-amino-3,4-dihydroquinazoline analogs (Figure 1 and Table 1). Inhibition of transport activity of all three hOCTs was perturbed, yielding IC_50_ estimates mostly in the low micromolar range, with a complete range from 0.5–47 μM (Table 1). Without exception, interaction (inhibition of transport activity) was poorest for hOCT2, yielding IC_50_ estimates from about 9–47 μM across the test compound set. The range of potencies at OCT1 was 1.3–14.3 μM. In contrast, OCT3 (0.5–12.2 μM) appeared to exhibit the greatest range in inhibition potencies, with A5MDQ (**7**) and A6FDQ (**11**) being among the most potent inhibitors. Specifically, the OCT3 inhibitory potency of the ADQ analogs decreased in the order of: 5-CH_3_ (**7**) = 6-F (**11**) > 5-Cl (**3**) > 8-Cl (**4**) = 6-Br (**10**) ≅ 6-CH_3_ (**6**) > 8-CH_3_ (**9**) ≅ 6-Cl (**1**) > 7-Cl (**2**) > 7-CH_3_ (**8**) > H (**5**). Although the range in potency was narrow (i.e., 24-fold), the unsubstituted compound **5** was the least potent, and nearly all the compounds were more potent than the initial lead compounds **1** and **2**. Furthermore, the rank-order of potencies at OCT3 did not follow the same rank-order of potencies at either OCT1 or OCT2.

**Figure 1:**
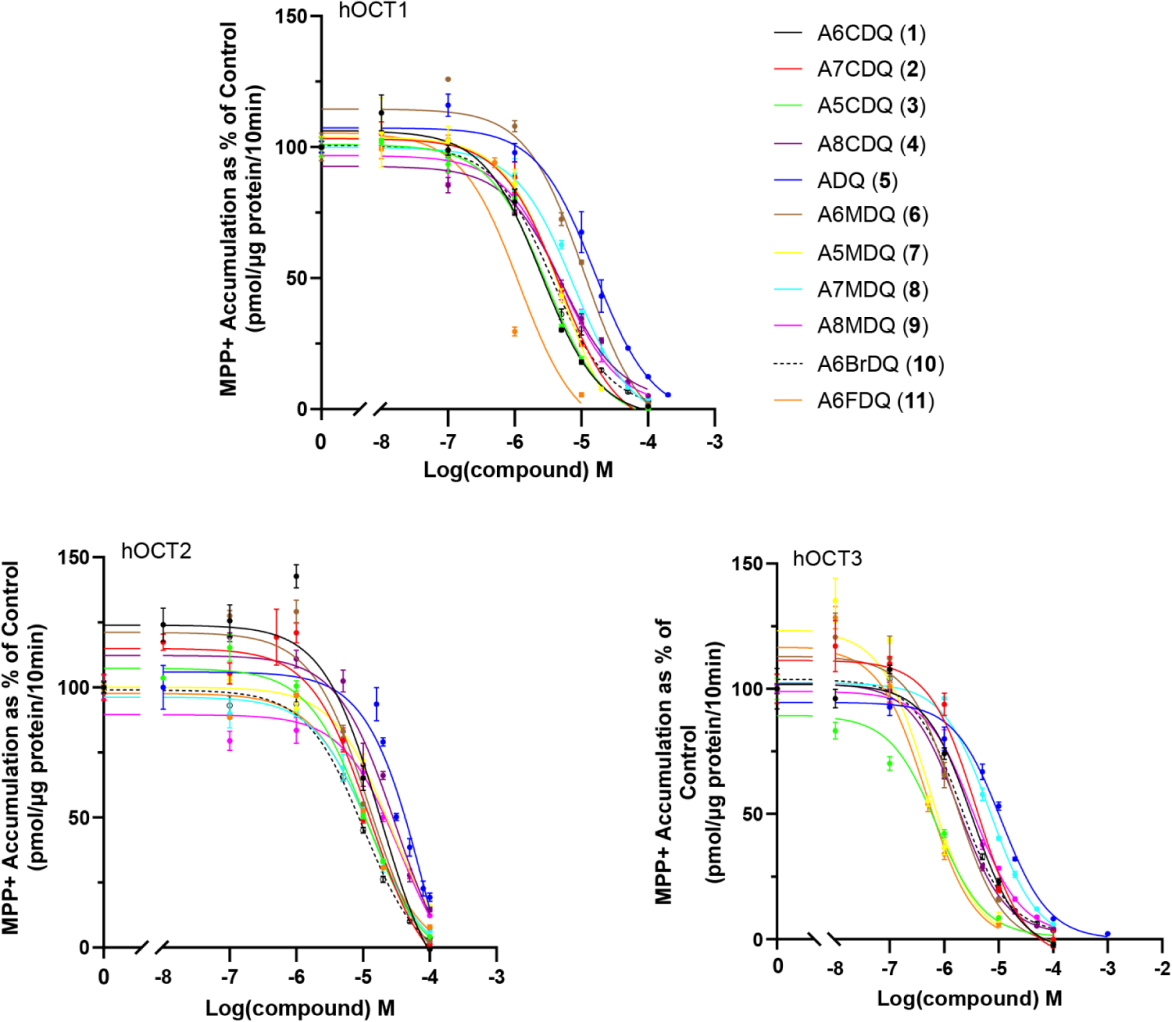
Kinetic assay. Representative plots showing uptake of MPP^+^ (at 1 μM for 1 min) measured in HEK293 cells stably expressing hOCT1, hOCT2 or hOCT3 in the presence of increasing concentrations (10^–8^ to 10^–3^ M) of unlabeled 2-amino-3,4-dihydroquinazoline analogs. All measurements were corrected for nonspecific background and are plotted as mean ± SEM (*n* = 2–3 per condition). The data were analyzed by nonlinear regression to determine the IC_50_ values reported in Table 1.

It is noted that some quinazoline analogs appeared to produce stimulation of hOCT-mediated MPP^+^ uptake at lower concentrations. However, the observed effects were not consistent within the hOCTs for individual analogs, but rather varied considerably, with no readily identifiable pattern or association with structural features. Similar inconsistent apparent stimulation of transporter activity at such low inhibitor concentrations has been reported in the literature, e.g., low concentrations of ciprofloxacin were observed to stimulate hOAT1, but not hOAT3 ^19^ and another group observed sparfloxacin acting as a “borderline stimulator” on MRP2.^20^ Indeed, in vitro stimulation of both SLC and ABC transporter activity has been reported for several drug classes including fluoroquinolones, steroids, anticancer chemotherapeutics, nonsteroidal anti-inflammatory drugs, and phenylguanidine analogs.^13,19–22^ While it has been suggested that this may be the result of interaction with allosteric binding site(s) this has not been directly examined,^20,21^ and whether such stimulatory effects on hOCTs (or any transporter) occur in vivo remains unknown.

### Modeling

OCT3 homology modeling and docking studies were performed using the cryo-EM structure of hOCT3 (PDB ID: 7ZHA)^12^ and quinazolines **1**–**11** reported here. All top-scoring (Gold2020/HINT) docking solutions from each quinazoline analog tested were oriented in a similar manner. Each nitrogen atom from the guanidine moiety was at the necessary angle and distance to form electrostatic interactions with the residues SER474, ASP478, and CYS477 (Figure 2). As demonstrated in Figure 3, the 5-chloro substituent of A5CDQ (**3**) (shown as a green space-filling sphere) fits snuggly into the pocket generated from the surrounding aromatic residues of PHE250, TRP223, TYR454, PHE32, and PHE36. Comparing the docking poses of A5CDQ (**3**) and A5MDQ (**7**) shows the possibility of the quinazoline analogs to bind in a rotameric fashion (Figure 3). The ring of A5MDQ (**7**) is likely flipped in relation to that of A5CDQ (**3**) due to enhanced face-to-face and edge-to-face interactions between the aromatic ring and the aromatic pocket where the 5-methyl substituent can only partake in hydrophobic interactions. However, the 5-chloro substituent of **3** can participate in electrostatic interactions as well as hydrophobic interactions, thus allowing binding modes to be flipped compared to **7**. Also shown in Figure 2 is a comparison of the docked quinazolines with the cryo-EM structure of decynium-22. There is little overlap between the top scoring solutions of ADQ analogs and decynium-22, the exception being the aryl substituents of the quinazoline analogs that occupy the same space as one of the aryl rings of decynium-22 in the binding pocket. In addition, the *N*-alkyl group from decynium-22 overlaps the aryl ring of the quinazolines.

**Figure 2:**
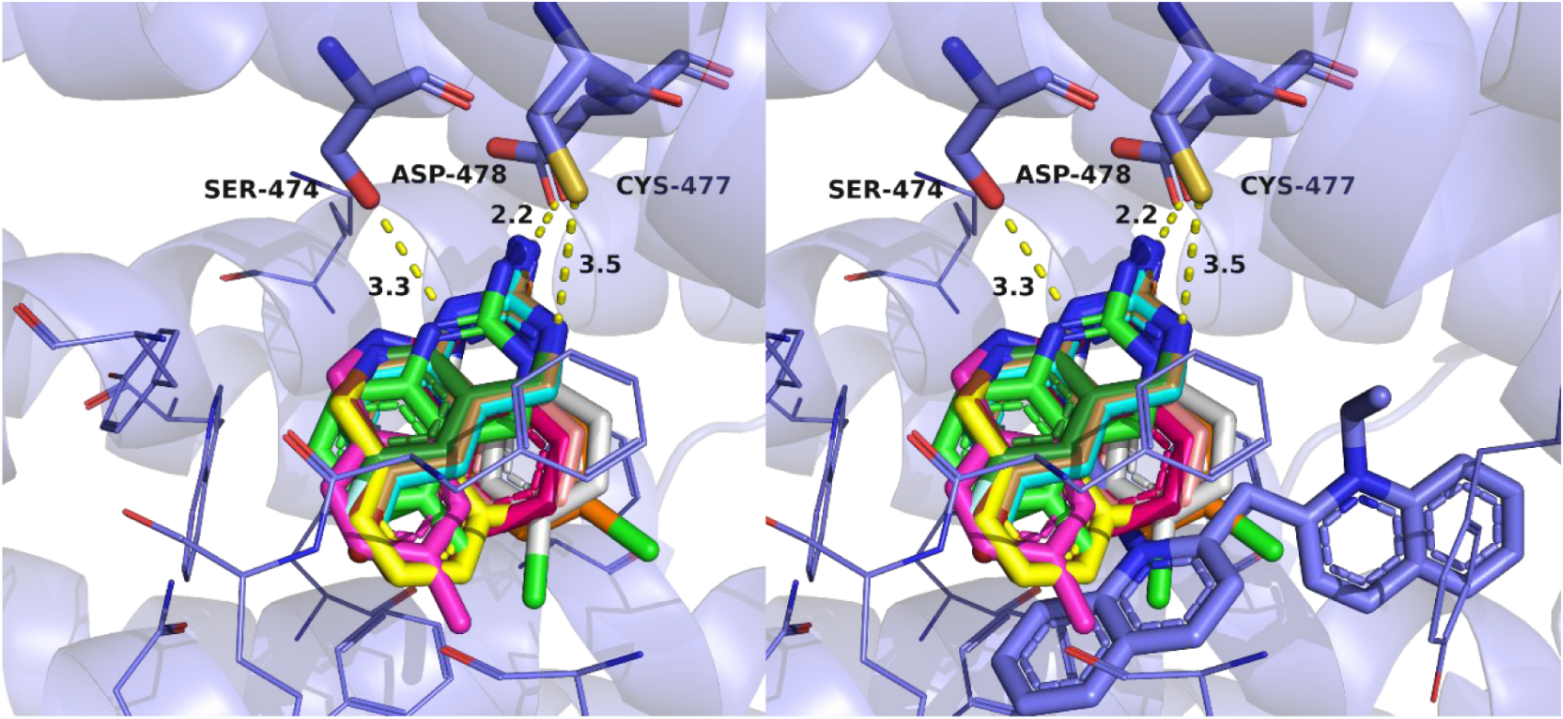
Binding mode of ADQ analogs at OCT3 (PDB ID: 7ZHA). A6CDQ (**1**; white), A7CDQ (**2**; orange), A5CDQ (**3**; peach), A8CDQ (**4**; brown), ADQ (**5**; pink), A6MDQ (**6**; cyan), A5MDQ (**7**; green), A7MDQ (**8**; magenta), A8MDQ (**9**; yellow), A6BrDQ (**10**; sand), and A6FDQ (**11**; marine) are shown as sticks. The amino acids in the binding pocket of OCT3 are represented as purple lines. Electrostatic interactions are shown as dashed yellow lines with interacting residues shown as purple sticks. Decynium-22 from the PDB structure is shown on the right as purple sticks for reference.

**Figure 3:**
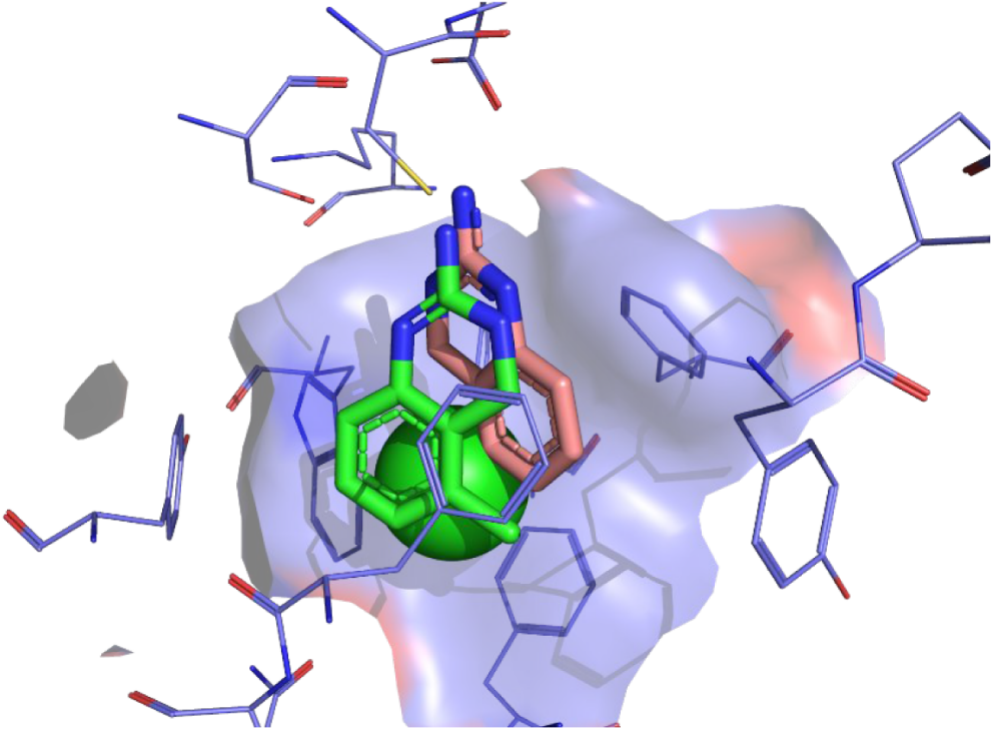
Rotameric binding modes of A5CDQ (**3**; peach) and A5MDQ (**7**; green) rendered as sticks at OCT3 (PDB ID: 7ZHA). The chloro substituent of A5CDQ is shown as a green space-filling sphere. The binding pocket of OCT3 is represented as purple lines with a surface to demonstrate how well the space filling sphere fits into an aromatic portion of the pocket.

### Mouse Tail Suspension Test (TST)

We previously reported that both A6CDQ (**1**)^14^ and A7CDQ (**2**)^15^ produce antidepressant-like effects in the mouse TST. To determine if the chloro group or its position is important for antidepressant-like action, we examined the 5- and 8-chloro substituted 2-aminodihydroquinazolines (A5CDQ; **3** and A8CDQ; **4**), as well as their parent aryl-unsubstituted analog ADQ (**5**). Also examined were A6MDQ (**6**) and A6FDQ (**11**). The 6-methyl-substituted analog, A6MDQ (**6**), had nearly comparable inhibitory potency at hOCT3 as A6CDQ (**1**) and was tested to determine if it retained the antidepressant-like action shown by A6CDQ (**1**) (i.e., is activity specifically related to the chloro substituent?). Compound A6FDQ (**11**) was examined because of its potency in the OCT3 assay, and ADQ (**5**) was examined to determine if the parent structure would show such activity (i.e., it displayed the lowest potency in the OCT3 assay).

A5CDQ (**3**), a positional isomer of the lead A6CDQ (**1**), was found to be active in the mouse TST (Figure 4). A5CDQ (**3**) significantly lowered the immobility time (22.60 ± 8.92 s) as compared to saline (88.99 ± 10.93 s) at a dose of 1 mg/kg similar to A6CDQ (**1**) and A7CDQ (**2**), but resulted in a U-shaped concentration–response curve. The effective dose for the 5-Cl analog, A5CDQ (**3**) was determined to be 0.57 mg/kg, ∼2.5-fold lower than that for the lead compound, A6CDQ (**1**), (ED_50_ = 0.23 mg/kg).^14^

**Figure 4:**
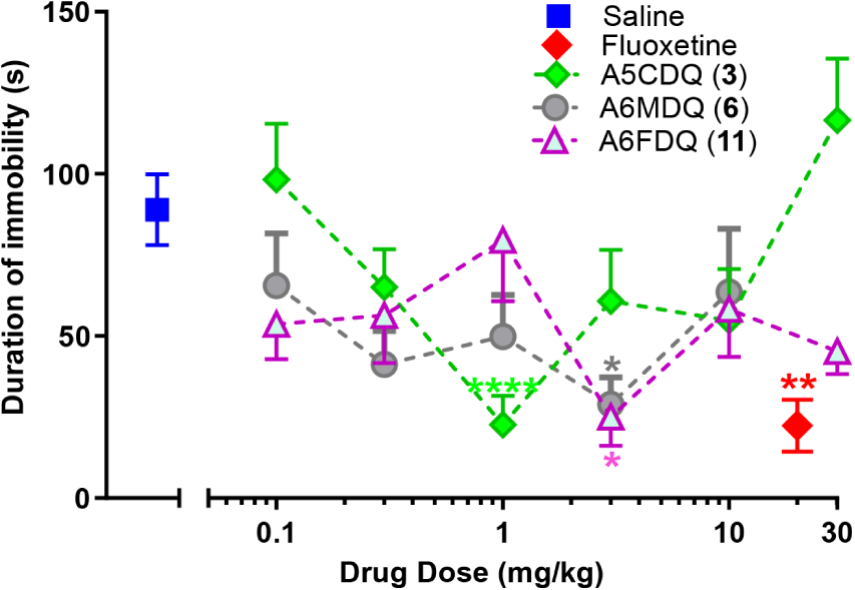
Effect (±SEM) of A5CDQ (**3**), A6MDQ (**6**) and A6FDQ (**11**) on duration of immobility in the mouse TST. All three ADQ analogs significantly reduced the duration of immobility at a dose of 1.0 (A5CDQ; **3**) and 3.0 mg/kg (A6MDQ; **6** and A6FDQ; **11**) as compared to saline according to one-way analysis of variance (ANOVA). i.e., A5CDQ (**3**) (F_7,63_ = 5.529, *p* = 0.0001), Dunnett’s post-hoc test (**** *p* < 0.0001), A6MDQ (**6**) (F_6,55_ = 3.144, *p* = 0.0101), Dunnett’s post-hoc test (* *p* < 0.05), A6FDQ (**11**) (F_7,66_ = 3.197, *p* = 0.0056), Dunnett’s post-hoc test (** *p* < 0.01), and fluoxetine (F_5,50_ = 3.714, *p* = 0.0062).

A6MDQ (**6**) was active in the mouse TST albeit at a slightly higher dose of 3 mg/kg (28.93 ± 8.32 s; Figure 4). A6FDQ (**11**) was also found to be active in the mouse TST and significantly lowered immobility times (25.02 ± 8.88 s) at 3 mg/kg when compared to saline (Figure 4), but here, too, also resulted in a U-shaped curve.

Both A8CDQ (**4**) and ADQ (**5**) at doses (0.1–30 mg/kg) failed to display antidepressant-like activity in the mouse TST (Figures S-1 and S-2). The commercially available SSRI, FLX was employed as a standard. FLX is commonly used in the TST at a dose of 20 mg/kg.^23^ We examined five doses of FLX and showed that it was inactive at a dose of <20 mg/kg.^15^

Overall, then, the ADQ analogs A5CDQ (**3**), A6MDQ (**6**) and A6FDQ (**11**) displayed antidepressant-like activity in the mouse TST (Figure 4) with a duration of immobility comparable to FLX (22.36 ± 8.06 s) but at a 6- to 20-fold lower dose.

### Locomotor Activity (LA) Assay

To eliminate the possibility that the antidepressant-like duration of immobility observed in the mouse TST resulted from a locomotor stimulant effect, we examined the active doses of A5CDQ (**3**), A6MDQ (**6**) and A6FDQ (**11**) identified in the mouse TST in a locomotor activity assay. The mice were injected intraperitoneally with 1.0 mg/kg of A5CDQ (**3**) or 3.0 mg/kg of A6MDQ (**6**) and A6FDQ (**11**) or saline and their locomotor activity was measured for a period of 45 min. The test duration for the locomotor activity includes the 30 min pretreatment time of the mouse TST, the duration of the mouse TST (6 min) and the 9 min after the mice were no longer suspended by their tails. Using a TruScan apparatus, 11 separate measures of locomotor action were examined (Figures S-3–S-6). Of the three 2-aminodihydroquinazoline analogs tested, A5CDQ (**3**), A6MDQ (**6**) and A6FDQ (**11**) at the TST-active doses, no statistically significant changes were observed for movement time (s), movement distance (cm) and ambulatory velocity (cm/s) when compared to saline (Figure S-3). Whereas no change in the number of movement episodes was observed for A5CDQ (**3**) and A6MDQ (**6**), a statistically significant (unpaired two-tailed *t* test) increase for A6FDQ (**11**) compared to saline was observed. Although the number of center entries and the amount of time spent in the center was reduced for mice treated with A6MDQ (**6**), the difference was not statistically significant compared to saline (Figure S-4). No differences were observed for center entries, center distance (cm) and center time (s) for A5CDQ (**3**) and A6FDQ (**11**) when compared to saline (Figure S-4). Additionally, the parameters – margin distance (cm) and margin time (s) – were similar for the drugs tested and saline (Figure S-5). Increased anxiety could account for reduced immobility times in mouse TST. However, no changes in the amount of time spent in the center vs the margin eliminated an anxiogenic-like effect (Figures S-4 and S-5). Decreases in both jumps and V-plane entries were observed for all three analogs A5CDQ (**3**; 1.0 mg/kg), A6MDQ (**6**; 3.0 mg/kg) and A6FDQ (**11**; 3.0 mg/kg). These decreases, however, were not statistically significant compared to saline (Figure S-6).

## Discussion

We have previously identified chloro analogs A6CDQ (**1**) and A7CDQ (**2**) as novel OCT inhibitors.^15^ To better understand the role and position of the chloro substituent, we examined the remaining two chloro-substituted analogs (i.e., **3** and **4**), and the possible contribution of the lipophilic and electronic properties of the chloro group to the inhibitory potency at hOCT1–3. Chloro and methyl substituents possess relatively similar size and lipophilic character but differ in their electronic nature; consequently, we considered the corresponding methyl analogs **6**–**9**. A methyl group possesses similar lipophilic (π = 0.71 and 0.56 for chloro and methyl, respectively) but opposite electronic (σ = 0.23 and −0.17 for chloro and methyl, respectively) character.^24^ If the electronic property of the chloro substituent of ADQ analogs contributes to OCT3 inhibition, then their methyl analogs should be less active compared to their chloro counterparts. Hence, a major goal was to probe the SAR of the aminodihydroquinazolines, with a focus on OCT3, to determine if the chloro groups of **1** and **2** are positionally required for their action, and to compare relative potencies at the three OCT subtypes. Consequently, a curated series of compounds was examined at each of the OCTs. It was found that all analogs displayed activity at all three transporters. However, at each OCT, the parent, unsubstituted compound (i.e., ADQ; **5**) was the least potent. At OCT3, the chloro group could be relocated to the 5- or 8-position (i.e., A5CDQ and A8CDQ, **3** and **4** respectively) with a slight improvement in inhibitory potency. Replacement of each chloro group by a methyl group had little effect (i.e., about a 2-fold increase or decrease for **6**, **7**, **8**, and **9**) on potency. The potency of the bromo and fluoro counterparts of A6CDQ (**1**), A6BrDQ (**10**) and A6FDQ (**11**), were found to be 2-fold and 8-fold higher than **1** at OCT3. The bromo substituent possesses the same electronic character as a chloro substituent and is slightly more lipophilic (π_Br_= 0.86); however, the fluoro substituent is less lipophilic (π_F_ = 0.14) but displays different electronic character (σ_F_ = 0.06).^24^ Additional analogs will require investigation before a role for the lipophilicity and/or electronic character of 6-position substituents can be defined, but it is evident that the OCT3 inhibitory action of **1** and **2** are not dependent upon the position of the chloro group. All compounds displayed somewhat similar potencies at OCT1 as compared to OCT3, but their potencies at OCT2 were generally lower.

Because OCT2 and OCT3 are located in neurons, it was of interest to determine if ADQ potencies covaried upon introduction of their various substituent groups, particularly where Gebauer et al.,^25^ demonstrated upon examination of 264 substances, that OCT3 transport had significantly greater overlap with OCT2 than OCT1, and that selectivity among the three OCTs varied with minor substituent alterations rather than with the general scaffolds. A simple comparison of pIC_50_ values of compounds in Table 1 for inhibition of OCT3 versus OCT2 (r = 0.255, 95% confidence interval (−0.4073 to 0.7414), *n* = 11, P (two-tailed) = 0.449) suggested that substituents have a different effect on potency at these two transporters. Indeed, OCT2 shares 49.73% identity to OCT3 (Table S-1), and A6FDQ (**11**) and A5MDQ (**7**) already display approximately 25- to 50-fold selectivity for OCT3 versus OCT2; hence, our work lays the groundwork for developing other ADQ analogs in the future that can be optimized to achieve greater selectivity. In support of this concept, others have reported that OCT selectivity can be achieved. For example, corticosterone (IC_50_ = 0.29 μM) displays selectivity for OCT3 relative to OCT1 (75-fold) and OCT2 (100-fold), and O-methylisoprenaline (metaprinaline) (IC_50_ = 4.38 μM), an isoprenaline metabolite, is OCT3-selective.^26^ In fact, Chen et al.^3^ have suggested that OCT3 is highly druggable from their HTS investigation of a compound library consisting of 2,556 prescription drugs, bioactive, and natural products, that identified 210 OCT3 inhibitors.

To support our findings from the biochemical and in vivo behavioral assays, we leveraged docking studies to probe the molecular interactions of ADQ analogs at the atomic level with OCT3. The three interactions with the residues SER474, ASP478, and CYS477 seem to have the greatest effect on the position and orientation of the quinazoline class of molecules at OCT3. The 1-position and the 3-position of the quinazoline analogs are likely able to flip, switching interactions with SER474 and CYS477 in a rotameric binding fashion. Analogs with a 5-position substituent were some of the most potent examined (Table 1) likely due to the favorable interactions with the aromatic residue pocket comprised of PHE250, TRP223, TYR454, and PHE32. In a comparison of decynium-22 with the quinazoline analogs (Figure 2), the minimal overlap of the two classes of agents (decynium-22 and ADQ analogs) demonstrates the unique binding modes of the quinazoline compounds as well as the extensive size of the OCT3 binding pocket. Demonstrated by the two currently solved structures of decynium-22 and corticosterone bound in the binding pocket of OCT3 (PDB ID: 7HZA and 7HZ6),^12^ OCT3 ligands can bind in a dissimilar manner yet also contain overlapping binding features. The current study suggests that ADQ analogs bind in a dissimilar manner yet share some overlapping binding features reported for decynium-22 and corticosterone. That is, ADQ analogs are unique and structurally different than decynium-22 or corticosterone, and it might be noted that decynium-22, a quaternary amine, interacts with multiple transporter family members due to its polyspecific nature and, thus, is “less clean as to absolute cause and effect for a particular transporter”,^2^ and the somewhat more OCT3-selective corticosterone is a steroid hormone with multiple actions including glucocorticoid and mineralocorticoid activity. In addition, the ADQ analogs are only about half the size of decynium-22 and corticosterone indicating that expanding the quinazoline scaffold is feasible and that it might be possible to exploit auxiliary interactions with binding pocket residues to obtain improved activity and selectivity. Future studies on increasing the size of the quinazoline substituents to find the extent of bulk tolerance for the large binding pocket of OCT3 may be beneficial in increasing activity and selectivity. Yet another avenue to achieve selectivity for OCT3 over OCT1 and OCT2 might be through a structure-based design campaign utilizing models of the solved cryo-EM structures of OCT1 and OCT2.^27^

Because (i) certain antidepressants are believed to act as SERT reuptake inhibitors but are also weak inhibitors of OCT3 (IC_50_ *ca* 30 to >500 μM),^5^ (ii) our compounds (e.g., A7CDQ; **2**) have already demonstrated insignificant involvement of the serotonin transporter (SERT; **2**, *K*_i_ > 10,000 nM, *K*_m_ = 43.6 μM),^14,15^ and (iii) OCT3 inhibitors have been suggested to display antidepressant like action,^5^ it was of interest to determine if the action of **1** and **2** in the TST was related to their specific structures (i.e., the presence and location of their chloro substituents), or if any other ADQ analogs might produce this effect. Hence, several were examined in the mouse TST; specifically, ADQ (**5**), A5CDQ (**3**), A8CDQ (**4**), A6MDQ (**6**), and A6FDQ (**11**) were evaluated in this assay. The parent compound, ADQ (**5**) and the 8-chloro positional isomer of **1** (i.e., **4**) were inactive at the doses examined. However, **3**, **6**, and **11** were active. It might be concluded, then, that the presence of a 6- or 7-chloro substituent is not essential for this action, and can be replaced with certain other substituents. These mouse studies should translate well to humans due to hOCT3 and mOCT3 sharing 86.57% identity (Table S-1) and both proteins contain the same three amino acid residues found from modeling to be important for the binding of the ADQ analogs (Figure S-7).

To ensure that the observed TST effect is not hampered by central stimulant activity we examined A5CDQ (**3**), A6MDQ (**6**) and A6FDQ (**11**) in the mouse locomotor activity assay. The results indicated an overall lack of locomotor stimulant effect for all three 2-aminodihydroquinazoline analogs at the doses examined. However, a statistically significant (unpaired two-tailed *t* test) increase for A6FDQ (**11**) compared to saline was observed. A single movement episode encompasses the period when the movement is initiated by the rodent and ends when the rodent stops moving. Locomotor stimulants decrease the rodent’s tendency to stop and hence decreases the number of movement episodes. But, this is ordinarily accompanied by a concomitant increase in movement distance (cm) and time (s). Since we observed an increase in movement episodes without an accompanying increase in movement distance (cm) and movement time (s) for A6FDQ (**11**), it might be concluded that the antidepressant-like effect observed for A5CDQ (**3**), A6MDQ (**6**) and A6FDQ (**11**) in the mouse TST were unlikely due to simple central stimulant effects.

In conclusion, only the presence and not necessarily the position of the chloro group of **1** and **2** was found to contribute to OCT inhibitory action. Specifically, for OCT3, 5-Cl > 8-Cl > 6-Cl > 7-Cl > H. Furthermore, it was demonstrated that the chloro group could be replaced by a methyl group, and that the 6-chloro of A6CDQ (**1**) could be replaced by a bromo or fluoro substituent as found in A6BrDQ (**10**) and A6FDQ (**11**) without loss of OCT3 action. Because **1** and **2** were previously found to be active in the mouse TST^15^ several of the new compounds were selected for evaluation: the 5-Cl and 8-Cl positional isomers of **1** (i.e., **3** and **4**), the parent compound ADQ (**5**), and two A6CDQ (**1**) analogs bearing a 6-position substituent other than chloro (i.e., **6** and **11**). The lack of action by **5** might be related to its low potency in the in vitro OCT3 assay; however, the lack of action by **4** is not readily explicable (e.g., pharmacokinetics, in vivo stability?). Compounds **3**, **6**, and **11** were active in the TST, indicating that the in vivo action of **1** and **2** are not solely limited to a chloro substituent at the aryl 6- or 7-position and that other substituents (e.g., methyl or fluoro) retain such activity. Our work has successfully furnished pharmacological tools that can be employed to study the underexplored OCTs. Future studies will focus on extending the SAR by examining additional substituents, to explore the nature of lipophilic/electronic character and bulk tolerance, to improve OCT3 versus OCT2 selectivity.

## Methods

### Synthesis

Compounds were characterized using a combination of melting point (mp), proton nuclear magnetic resonance (^1^H NMR), infrared (IR), and mass (MS) spectrometry. Purity of the compounds was determined based on elemental analysis for C, H and N performed by Atlantic Microlab Inc. (Norcross, GA) and compounds were considered pure if the values obtained were within 0.4% of theoretical values. A MEL TEMP melting point apparatus was utilized to obtain uncorrected melting points (mp) of compounds in glass-walled capillary tubes. ^1^H NMR spectra were obtained either using a Bruker ARX 400 MHz or Bruker AVANCE III 400 MHz spectrometer. The spectra obtained were reported by indicating the position of the signals in parts per million (ppm) downfield from tetramethylsilane (TMS), used as an internal standard, followed by the splitting pattern of the signal (s = singlet, d = doublet, t = triplet, q = quartet, dd = doublet of doublets, m = multiplet), coupling constant (*J*, Hz) and integration. IR spectra were determined using Thermo Nicolet iS10 FT-IR. MS were obtained using a Waters Acquity TQD (tandem quadrupole) spectrometer utilizing electrospray ionization in positive ion mode. Reactions were followed using thin-layer chromatography (TLC) on silica gel GHLF plates (250 μm, 2.5 × 10 cm; Analtech Inc. Newark, DE). For the purposes of biological studies water-soluble salts of compounds (hydrochloride, or hydrobromide) were prepared except for 2-amino-5-methyl-3,4-dihydroquinazoline (**7**) for which a suitable salt could not be obtained because the salts obtained were hygroscopic. Compound **7** was prepared as a concentrated stock in DMSO and diluted in transport buffer to a final concentration ≤0.1%, control cells were exposed to 0.1% DMSO.

#### 2-Amino-6-methyl-3,4-dihydroquinazoline Hydrochloride (**6**)

BH_3_·THF complex (1 M in THF, 6 mL, 6.0 mmol) was added in a dropwise manner at 0 °C to 2-amino-6-methylquinazolin-4­(3*H*)-one (**14**) (0.28 g, 1.6 mmol) under an N_2_ atmosphere. The stirred reaction mixture was heated at reflux for 1 h to give a clear solution, allowed to cool to room temperature, quenched by addition of HCl (6 N, to pH 3). The reaction mixture was then heated to evaporate THF until THF could no longer be detected by smell. The residue was cooled to 0 °C (ice-bath) and a solution of NaOH (15%, to pH 12) was added. Hot CHCl_3_ (3 × 10 mL) was used to extract the desired compound, which settled between H_2_O and CHCl_3_ layers. The solid obtained was collected by filtration and washed with cold CHCl_3_ to afford 0.15 g (44%) of the free base of **6** as a white solid: mp > 300 °C. A saturated solution of gaseous HCl in EtOH was added to a solution of the free base of **6** in EtOH until the solution became acidic (pH = 3). The EtOH was removed under reduced pressure to obtain a crude, white solid which upon crystallization from EtOH yielded 0.04 g (23%) of **6** as off-white crystals: mp 172–175 °C. ^1^H NMR (DMSO-*d*_6_) δ 2.25 (s, 3H, CH_3_), 4.46 (s, 2H, CH_2_), 6.86–6.88 (d, *J* = 8 Hz, 1H, ArH), 6.99 (s, 1H, ArH), 7.05–7.07 (d, *J* = 8 Hz, 1H, ArH), 7.54 (s, 1H, NH D_2_O ex), 8.46 (s, 1H, NH D_2_O ex), 10.74 (s, 1H, NH D_2_O ex). Anal. Calcd for (C_9_H_11_N_3_·1.1HCl) C, 53.70; H, 6.06; N, 20.87. Found: C, 53.95; H, 6.06; N, 20.84.

#### 2-Amino-5-methyl-3,4-dihydroquinazoline (**7**)

Cyanogen bromide (3 M solution in CH_2_Cl_2_, 2 mL, 6.2 mmol) was added to a stirred solution of 2-(aminomethyl)-3-methylaniline (**19**) (0.56 g, 4.1 mmol) in toluene (6 mL). The stirred reaction mixture was heated at reflux overnight to give a dark brown suspension, then allowed to cool to room temperature followed by removal of toluene under reduced pressure. The residue was dissolved in H_2_O (20 mL) followed by addition of a saturated solution of NaHCO_3_ resulting in precipitation of a yellow solid. The solid was collected by filtration to afford 0.07 g (11%) of **7** as a light-brown solid: mp 180–184 °C. ^1^H NMR (DMSO-*d*_6_) δ 2.07 (s, 3H, CH_3_), 4.33 (s, 2H, CH_2_), 6.52–6.50 (d, *J* = 8 Hz, 1H, ArH), 6.62–6.60 (d, *J* = 8 Hz, 1H, ArH), 6.93–6.89 (t, *J* = 8 Hz, 1H, ArH). HRMS (M^+^+H) calcd. for C_9_H_12_N_3_ 162.1005; found 162.1045.

#### 2-Amino-7-methyl-3,4-dihydroquinazoline Hydrochloride (**8**)

Cyanogen bromide (3 M solution in CH_2_Cl_2_, 3 mL, 9.4 mmol) was added to a stirred solution of 2-(aminomethyl)-5-methylaniline (**20**) (0.85 g, 6.2 mmol) in toluene (6 mL). The stirred reaction mixture was heated at reflux overnight, allowed to cool to room temperature followed by removal of toluene under reduced pressure. The residue was dissolved in H_2_O (80 mL) followed by addition of a saturated solution of NaHCO_3_ to pH = 9 to 10. The aqueous layer was further basified by addition of NaOH (3 N, to pH 12). The solid was collected by filtration to afford 0.63 g of crude free base of **8**. The solid was dissolved in MeOH (10 mL) and a saturated solution of gaseous HCl in EtOH was added (pH 1–2) and the reaction mixture was allowed to stir at room temperature overnight. The MeOH was removed under reduced pressure to afford 0.60 g (79%) which upon recrystallization from EtOH gave 0.20 g (26%) of **8** as a white solid: mp 204–205 °C. ^1^H NMR (DMSO-*d*_6_) δ 2.24 (s, 3H, CH_3_), 4.41 (s, 2H, CH_2_), 6.75 (s, 1H, ArH), 6.86–6.89 (d, *J* = 12 Hz, 1H, ArH), 7.03–7.05 (d, *J* = 8 Hz, 1H, ArH), 7.60 (s, 2H, NH_2_), 8.46 (s, 1H, NH), 10.77 (s, 1H, NH). Anal. Calcd for (C_9_H_11_N_3_·1.1HCl) C, 53.70; H, 6.06; N, 20.87. Found: C, 53.72; H, 5.97; N, 21.19.

#### 2-Amino-8-methyl-3,4-dihydroquinazoline Hydrochloride (**9**)

Cyanogen bromide (3 M solution in CH_2_Cl_2_, 3 mL, 9.9 mmol) was added to a stirred solution of 2-(aminomethyl)-6-methylaniline (**21**) (0.90 g, 6.6 mmol) in toluene (6 mL). The stirred reaction mixture was heated at reflux overnight and then allowed to cool to room temperature. This was followed by removal of toluene under reduced pressure to give a sticky orange-pink solid. The residue was dissolved in H_2_O (40 mL) followed by addition of a saturated solution of NaHCO_3_ to pH = 9 to 10. The aqueous portion was further basified by addition of NaOH (3 N, to pH 12). The solid was collected by filtration and washed with H_2_O (30 mL) to afford 0.49 g of crude free base of **9**. The free base was dissolved in EtOH (20 mL) and a saturated solution of gaseous HCl in EtOH was added (pH 1–2) and the reaction mixture was allowed to stir at room temperature overnight. EtOH was removed under reduced pressure to afford 0.40 g of a white solid which upon recrystallization from EtOH gave 0.21 g (16%) of **9** as a white solid: mp 168–170 °C. ^1^H NMR (DMSO-*d*_6_) δ 2.28 (s, 3H, CH_3_), 4.47 (s, 2H, CH_2_), 6.09–7.02 (q, *J* = 6 Hz, 2H, ArH), 7.10–7.12 (q, *J* = 9 Hz, 1H, ArH), 7.85 (s, 2H, NH_2_), 8.54 (s, 1H, NH), 10.01 (s, 1H, NH). Anal. Calcd for (C_9_H_11_N_3_·1HCl) C, 54.69; H, 6.12; N, 21.26. Found: C, 54.51; H, 6.06; N, 21.13.

#### 2-Amino-6-bromo-3,4-dihydroquinazoline Hydrobromide (**10**)

Cyanogen bromide (3 M solution in CH_2_Cl_2_, 2 mL, 6.0 mmol) was added to a stirred solution of 2-(aminomethyl)-4-bromoaniline (**22**) (0.80 g, 4.0 mmol) in toluene (6 mL). The stirred reaction mixture was heated at reflux overnight and allowed to cool to room temperature. This was followed by removal of toluene under reduced pressure to give a sticky, yellow solid. The residue was washed with H_2_O (50 mL) and the solid was collected by filtration and washed with H_2_O (30 mL) to afford 0.40 g (32%) of **10** as a beige solid: mp 196–198 °C. ^1^H NMR (DMSO-*d*_6_) δ 4.49 (s, 2H, CH_2_), 6.95–6.97 (d, *J* = 8 Hz, 1H, ArH), 7.44–7.46 (t, *J* = 4 Hz, 2H, ArH), 7.57 (s, 2H, NH_2_), 8.30 (s, 1H, NH), 10.52 (s, 1H, NH). Anal. Calcd for (C_8_H_8_N_3_Br·0.8HBr) C, 33.04; H, 3.05; N, 14.45. Found: C, 33.23; H, 3.00; N, 14.35.

#### 2-Amino-6-fluoro-3,4-dihydroquinazoline Hydrochloride (**11**)

2-Amino-6-fluoroquinazolin-4­(3*H*)-one (**25**) (0.50 g, 2.8 mmol) was added in a portionwise manner to a stirred solution of BH_3_·THF complex (1 M in THF, 11 mL, 11.0 mmol) at 0 °C (ice-bath) under an N_2_ atmosphere. To this, anhydrous THF (16 mL) was added. The stirred reaction mixture was heated at reflux for 1.2 h, allowed to cool to room temperature, quenched by addition of HCl (6 N, to pH 2), and then heated at reflux for 35 min. The reaction mixture was allowed to cool to room temperature and basified with NaOH (15% as well as NaOH pellets, to pH 12). THF was evaporated under reduced pressure to give a white precipitate which was collected by filtration and upon recrystallization from EtOH/Et_2_O afforded 0.09 g (%) of **11** as a cream-colored solid: mp 228–230 °C ^1^H NMR (DMSO-*d*_6_) δ 4.49 (s, 2H, ArCH_2_), 6.99–7.02 (m, 1H, ArH), 7.09–7.13 (m, 2H, ArH), 7.68 (s, 2H, NH_2_), 8.55 (s, 1H, NH), 10.96 (s, 1H, NH). HRMS (ESI-TOF) calcd for C_8_H_9_N_3_F [M + H] 166.0775, found 166.0761.

#### 6-Methylisatoic Anhydride (**13**)

Urea-H_2_O_2_ complex (0.78 g, 8.3 mmol) and 3 drops of H_2_SO_4_ were added to a solution of 5-methylisatin (**12**) (1.00 g, 6.2 mmol) in 8 mL of HCOOH. The reaction mixture was then sonicated for 35 min with occasional stirring. The urea-hydrogen peroxide complex was prepared according to a reported procedure^28^ by adding urea (4.00 g, 66.6 mmol) portion wise to H_2_O_2_ (30% aq., 11.32 mL) heated to 55 °C and keeping the temperature below 60 °C. The reaction mixture was poured on to an evaporating dish and left overnight. The solid obtained was collected by filtration to afford 4.87 g (78%) of the urea-H_2_O_2_ complex as a white solid. The precipitate formed during sonication of reaction mixture was collected by filtration and washed with Et_2_O to yield 0.87 g of a bright yellow-orange solid which upon recrystallization from HOAc gave 0.62 g (56%) of **13** as bright orange crystals: mp 227–230 °C (lit^18^ mp 233 °C).

#### 2-Amino-6-methylquinazolin-4­(3*H*)-one (**14**)

S-Methylisothiouronium sulfate (0.86 g, 3.1 mmol) and Na_2_CO_3_ (0.36 g, 3.4 mmol) were added to a solution of 6-methylisatoic anhydride (**13**) (0.40 g, 2.2 mmol) in 80% aq. MeCN (12 mL). The reaction mixture was heated at reflux for 2.5 h. The reaction mixture was then allowed to cool to room temperature and the precipitate formed was collected by filtration and washed with 80% aq. MeCN to yield 0.18 g (46%) of **14** as a pale white solid: mp > 300 °C (lit^29^ mp >250 °C).

#### 2-(Aminomethyl)-3-methylaniline (**19**)

A complex of BH_3_·THF (1 M in THF, 30 mL, 30.3 mmol) was added in a dropwise manner at 0 °C (ice-bath) to a stirred solution of 2-amino-6-methylbenzonitrile (**15**) (1.00 g, 7.6 mmol) in anhydrous THF (15 mL) under an N_2_ atmosphere. The stirred reaction mixture was heated at reflux overnight, allowed to cool to room temperature, quenched by addition of HCl (6 N, to pH 2), and heated at reflux for 30 min. The reaction mixture was allowed to cool to room temperature and then to 0 °C (ice-bath) and basified with NaOH (15% as well as NaOH pellets, to pH 12) and extracted with CH_2_Cl_2_ (4 × 20 mL). The combined organic portion was washed with brine (50 mL), dried (MgSO_4_) and evaporated under reduced pressure to afford 0.69 g (67%) of **19** as a sticky, brown solid. IR spectroscopy indicated the absence of a cyano band at 2222 cm^–1^ as found in the starting material.

#### 2-(Aminomethyl)-5-methylaniline (**20**)

A complex of BH_3_·THF (1 M in THF, 30 mL, 30.3 mmol) was added in a dropwise manner at 0 °C (ice-bath) to a stirred solution of 2-amino-4-methylbenzonitrile (**16**) (1.00 g, 7.6 mmol) in anhydrous THF (15 mL) under an N_2_ atmosphere. The stirred reaction mixture was heated at reflux overnight, allowed to cool to room temperature, quenched by addition of HCl (6 N, to pH 2), and refluxed for 35 min. The reaction mixture was allowed to cool to room temperature and then at 0 °C (ice-bath), was basified with NaOH (3 N, to pH 12) and extracted with CH_2_Cl_2_ (2 × 50 mL). The combined organic portion was washed with H_2_O (50 mL), dried (MgSO_4_) and evaporated under reduced pressure to afford 0.89 g (85%) of **20** as a sticky solid: mp 82–86 °C. IR spectroscopy indicated the absence of a cyano band at 2208 cm^–1^ as found in the starting material.

#### 2-(Aminomethyl)-6-methylaniline (**21**)

A complex of BH_3_·THF (1 M in THF, 30 mL, 26.2 mmol) was added in a dropwise manner at 0 °C (ice-bath) to 2-amino-3-methylbenzonitrile (**17**) (1.00 g, 7.6 mmol) under an N_2_ atmosphere. The stirred reaction mixture was heated at reflux overnight, allowed to cool to room temperature, quenched by addition of HCl (6 N, to pH 2), and heated at reflux for 30 min. The reaction mixture was allowed to cool to room temperature and then to 0 °C (ice bath) and basified with NaOH (3 N, to pH 12) and extracted with CH_2_Cl_2_ (2 × 25 mL). The combined organic portion was washed with H_2_O (50 mL), dried (MgSO_4_) and evaporated under reduced pressure to afford 0.92 g (89%) of **21** as a sticky solid. IR spectroscopy indicated the absence of a cyano band at 2219 cm^–1^ as found in the starting material.

#### 2-(Aminomethyl)-4-bromoaniline (**22**)

A complex of BH_3_·THF (1 M in THF, 20 mL, 20.3 mmol) was added in a dropwise manner at 0 °C (ice-bath) to 2-amino-5-bromobenzonitrile (**18**) (1.00 g, 5.1 mmol) under an N_2_ atmosphere. The stirred reaction mixture was heated at reflux for 2.5 h, allowed to cool to room temperature, quenched by addition of HCl (6 N, to pH 2), and heated at reflux for 30 min. The reaction mixture was allowed to cool to room temperature and then to 0 °C (ice bath) and basified with NaOH (3 N, to pH 12) and extracted with CH_2_Cl_2_ (3 × 25 mL). The combined organic portion was washed with H_2_O (2 × 100 mL), dried (MgSO_4_) and evaporated under reduced pressure to afford 0.81 g (79%) of **22** as a white waxy solid: mp 110–112 °C. IR spectroscopy indicated the absence of a cyano band at 2218 cm^–1^ as found in the starting material.

#### 2-Amino-6-fluoroquinazolin-4­(3*H*)-one (**25**)

S-Methylisothiourea sulfate (0.92 g, 3.3 mmol) and Na_2_CO_3_ (0.37 g, 3.5 mmol) were added to a stirred solution of 6-fluoroisatoic anhydride^18^ (**24**) (0.60 g, 3.3 mmol) in MeCN (80% aq., 10 mL). The stirred reaction mixture was heated at reflux for 1.5 h to give a green solution, then allowed to cool to room temperature. The solid obtained was collected by filtration and washed with 80% aq. MeCN (30 mL) to afford 0.53 g (89%) of **25** as a cream-colored solid: mp 370 °C. ^1^H NMR (DMSO-*d*_6_) δ 6.39 (s, 1H, NH), 7.22–7.26 (q, *J* = 4 Hz, 1H, ArH), 7.42–7.47 (m, 1H, ArH) 7.53–7.56 (dd, 1H, ArH). IR (solid, cm^–1^) 668, 693, 734, 791, 823, 886, 937, 1074, 1127, 1152, 1214, 1251, 1347, 1387, 1479, 1515, 1569, 1659, 2769, 3039, 3334.

### Molecular Modeling Studies

#### Protein Model

The cryo-EM structure of the OCT3 complex with decynium-22 (PDB ID: 7ZHA)^12^ was used as a template to generate a model for docking studies. To prepare the model, hydrogen atoms were added, and the protein was minimized using the Tripos Force Field with Gasteiger-Hückel charges within SYBYL X2.1.1. Decynium-22 was then removed and the protein was used in the docking study.

#### Docking Studies

Gold2020 docking suite was used for docking all compounds. First, compounds were sketched in SYBYL X2.1.1 and minimized using the Tripos Force Field with Gasteiger-Hückel charges. The binding pocket of the OCT3 model was defined by a 10-Å radius surrounding the essential amino acid residues ASP478.^30^ Docking was then performed generating 100 solutions for each compound.

#### Hydropathic Analysis

Each docking solution was then merged into the model protein, minimized, extracted, and Hydropathic INTeractions (HINT)^31^ analysis was performed using SYBYL 8.1 and SYBYL’s Programming Language (SPL) scripts.

## Biological Studies

### In Vitro

#### Cell Lines

Transporter expressing Human Embryonic Kidney 293 (HEK293) cell lines were generated using cationic lipid-based transfection. Briefly, 1 μg plasmid DNA (pcDNA3 base vector or pcDNA3 containing either hOCT1, hOCT2 or hOCT3) was combined with 2 μL Lipofectamine 2000 (Invitrogen), mixed, diluted in 100 μL Opti-MEM (Invitrogen) and applied to HEK293 cells at 50–60% confluency in 12-well plates (Corning Inc., Corning, NY). Fresh Dulbecco’s Modified Eagle’s Medium culture medium (DMEM high glucose) was applied just prior to the addition of transfection agents. After incubating for 24 h at 37 °C/5% CO_2_, the transfection medium was removed and replaced with fresh DMEM high glucose containing Geneticin (G418; 1 mg/mL) to select for successfully transfected cells for a period of 14–21 days. The generated HEK293-control (empty vector) and HEK293-hOCT(1–3) expressing cell lines were maintained in DMEM high glucose with 10% FBS, and 1% penicillin/streptomycin at 37 °C with 5% CO_2_ in 25 or 75 mm^2^ polystyrene flasks. Lines were maintained under antibiotic-selective pressure (250 μg/mL G418) and subcultured every 3–4 days.

#### hOCT Functional Assay

Assay conditions, MPP^+^ concentration and accumulation time were as previously reported.^13,15^ Briefly, 2 × 10^5^ cells/well of each cell line (HEK293 stably transfected with empty vector, hOCT1, hOCT2 or hOCT3) were plated in 24-well tissue culture plates and grown for 48 h without antibiotics. To initiate uptake assay, cells were equilibrated with transport buffer (500 μL of Hanks’ balanced salt solution containing 10 mM HEPES, pH 7.4) for 10 min after which the transport buffer was replaced with 500 μL of fresh transport buffer containing 1 μM unlabeled 1-methyl-4-phenylpyridinium (MPP^+^) spiked with [^3^H]­MPP^+^ (0.25 μCi/mL) in the absence or presence of the novel ADQ analogs (10^–8^ to 10^–3^ M). After 1 min incubation, cells were rinsed three times with ice-cold transport buffer, lysed with 200 μL of 1 M NaOH and then neutralized with 250 μL of 1 M HCl + 200 μL of 0.1 M HEPES. Sample radioactivity was quantified by liquid scintillation counting and normalized by total protein (Bradford method) such that uptake was calculated as picomoles of MPP^+^ per milligram total cell protein (after correction for background accumulation in empty vector transfected control cells) and is reported as percent of control. Each experiment was performed at least three times with triplicate wells for each concentration. Half maximal inhibitory concentrations (IC_50_) were calculated using nonlinear regression with GraphPad Prism Software version 10.6.0 (GraphPad Software, LLC).

### In Vivo

#### Drugs

Fluoxetine hydrochloride (Prozac, Batch 4*A*/80352; Eli Lilly) was purchased from Tocris. A5CDQ (**3**), A8CDQ (**4**), A6MDQ (**6**), and A6FDQ (**11**) were used as hydrochloride salts. A5CDQ (**3**) was available from previous studies for testing. A8CDQ (**4**), A6MDQ (**6**), and A6FDQ (**11**) were synthesized for the current study. Solutions of all compounds were prepared by dissolving in 0.9% saline and were administered via intraperitoneal (ip) injections in a total volume of 10 mL/kg body weight. The solutions were stored overnight in a refrigerator and were used over a three-day period.

#### Animals

Male ICR mice (20–24 g) from Harlan Laboratories Inc. (Indianapolis, IN) were used for the mouse TST. The animals were housed and maintained in solid-bottom plastic cages in a temperature (∼22 °C)- and humidity (∼50%)-controlled environment with a standard 12:12 h dark:light cycle. No restrictions were placed on food and water, which were available *ad lib.* After an acclimatization period of 2 days the experiments were conducted in accordance to IACUC protocol AM10399 with the standards set by the Institutional Animal Care and Use Committee (IACUC) of Virginia Commonwealth University (VCU) and the National Institutes of Health’s (NIH) Guide for the Care and Use of Laboratory Animals.

#### Mouse Tail Suspension Test (TST)

The experiment was conducted by following protocols previously established by our laboratory.^14^ Mice were brought to the room where the experiment was conducted at least 2 h prior to start of the experiment. Mice were marked and weighed 1 h prior to the experiment which was conducted between 1200 to 1700 h. Mice were suspended ∼1.5 cm from the tips of their tails from a bar at a distance of 60 cm from the bench using industrial grade Duct Tape (2 × 1/2 in.) after the pretreatment time. The experiments were conducted in a blind manner as the experimentor was unaware of the identity of the drug as well as dose being injected. The mice were injected with saline (10 mL/kg; 30 min pretreatment time), fluoxetine hydrochloride (20 mg/kg; 30 min pretreatment time), A5CDQ (**3**) (0.1, 0.3, 1.0, 3.0, 10, or 30 mg/kg; 30 min pretreatment time), A8CDQ (**4**) (0.1, 0.3, 1.0, 3.0, 10, or 30 mg/kg; 30 min pretreatment time), ADQ (**5**) (0.1, 0.3, 1.0, 3.0, 10, or 30 mg/kg; 30 min pretreatment time), A6MDQ (**6**) (0.1, 0.3, 1.0, 3.0, or 10 mg/kg; 30 min pretreatment time), A6FDQ (**11**) (0.1, 0.3, 1.0, 3.0, 10, or 30 mg/kg; 30 min pretreatment time), using a random number table.^32^ The mice were administered the compounds via intraperitoneal (ip) injections and tested only once, and each compound was tested in 8–11 mice (*n* = 8–11 mice/treatment). The experiment (duration of 6 min) was video recorded using a Canon Rebel T3i camera. The immobility times for each compound were determined by viewing each mouse’s 6 min video recording and using a stopwatch to record the immobility times. Each mouse was scored in triplicate and a mean of these scores was utilized. Immobility was defined as lack of movement or passive swaying. Mobility was defined as running motions, body jerks, movement of only hind or fore legs, or attempts to catch its own tail. The mice were sacrificed using a CO_2_ chamber at the end of the experiment.

#### Locomotor Activity (LA) Assay

The experiment was conducted by following protocols previously established by us.^14^ Mice were acclimated to the room where the experiment was conducted for at least 2 h prior to start of the experiment. Mice were marked and weighed 30 min prior to the experiment, which was conducted between 1000 to 1500 h. Mice were placed in TruScan locomotor activity chambers immediately after injections (0 min pretreatment time). The animals were injected with saline (10 mL/kg; 0 min preinjection time), A5CDQ (**3**) (1.0 mg/kg; 0 min preinjection time), A6MDQ (**6**) (3.0 mg/kg; 0 min preinjection time) or A6FDQ (**11**) (3.0 mg/kg; 0 min preinjection time) using a random number table.^32^ The mice were administered the compounds via intraperitoneal (ip) injection and tested only once, and each compound was tested in 9 mice (*n* = 9 mice/treatment). The experiment (duration of 45 min) was conducted by placing injected mice in TruScan activity system (Coulbourn Instruments, Allentown, PA) photocell arena chambers (model E63–10; 26 cm × 26 cm × 39 cm). Since the walls of the chamber were transparent black screens were placed between the individual chambers to prevent the mice from being influenced by each other. The movement of the mice were tracked by two infrared photodetectors and the information was collated using the accompanying software. After each run, the TruScan activity chambers were wiped down with a detergent solution and the plates washed and dried prior to the next run. The following parameters were analyzed: movement episodes, movement time (s), movement distance (cm), ambulatory velocity (cm/s), margin distance (cm), margin time (s), center distance (cm), center time (s), center entries, jumps and vertical plane (V-plane) enteries. The mice were sacrificed using a CO_2_ chamber at the end of the experiment.

#### Analysis

The data collected were analyzed using one-way analysis of variance (ANOVA) followed by Dunnett’s post-hoc test for the mouse TST and unpaired two-tailed *t* test for the locomotor activity assay in GraphPad Prism 10.6.01 (GraphPad Software Inc., La Jolla, CA). Outliers were identified using GraphPad QuickCalcs which utilized Grubb’s test.

## Supplementary Material



## Notes

Results for A8CDQ (**4**) and ADQ (**5**) in the mouse TST (Figures S-1 and S-2); mouse LA results for the active doses of A5CDQ (**3**), A6MDQ (**6**), and A6FDQ (**11**) (Figures S-3–S-6); sequence alignment of hOCT3, mOCT3, hOCT2, and hOCT1 (Figure S-7); percent identity matrix of hOCT3, mOCT3, hOCT2, and hOCT1 (Table S-1) (PDF)
