# Supplementary material for: The 2‑Amino-3,4-dihydroquinazoline Molecular Scaffold as Novel OCT3 Inhibitor
Source: ACS Chem Neurosci. 2026 Mar 20;17(7):1375–86. doi: 10.1021/acschemneuro.5c01026 (PMC13047533; doi:10.1021/acschemneuro.5c01026)
Supplement: Supplementary file 1 [file cn5c01026_si_001.pdf]

Supporting Information

The 2-amino-3,4-dihydroquinazoline molecular scaffold as novel OCT3 inhibitor

Kavita A. Iyer,[1] Xiaolei Pan,[2] Charles B. Jones,[1] Hebing Liu[2], Malaika Argade,[1] Osama I. Alwassil,[1] Douglas H. Sweet,[2] Małgorzata Dukat[1*]

[1]Department of Medicinal Chemistry, School of Pharmacy, Virginia Commonwealth University, Richmond, VA 23298

[2]Department of Pharmaceutics, School of Pharmacy, Virginia Commonwealth University, Richmond, VA 23298

TABLE OF CONTENTS

Content	Page
**Figure S-1.** Effect (± SEM) of A8CDQ (**4**) on duration of immobility in the mouse TST	S2
**Figure S-2.** Effect (± SEM) of ADQ (**5**) on duration of immobility in the mouse TST	S2
**Locomotor Activity Assay**	S3
**Figure S-3.** Effect (± SEM) of A5CDQ (**3**), A6MDQ (**6**) and A6FDQ (**11**) at 1.0, 3.0 and 3.0 mg/kg, respectively for movement episodes, movement time (s), movement distance (cm) and ambulatory velocity (cm/s) in the locomotor activity assay	S4
**Figure S-4.** Effect (± SEM) of A5CDQ (**3**), A6MDQ (**6**) and A6FDQ (**11**) at 1.0, 3.0 and 3.0 mg/kg, respectively for center entries, center distance (cm) and center time (s) in the locomotor activity assay	S5
**Figure S-5.** Effect (± SEM) of A5CDQ (**3**), A6MDQ (**6**) and A6FDQ (**11**) at 1.0, 3.0 and 3.0 mg/kg, respectively for margin distance (cm) and margin time (s)	S6
**Figure S-6.** Effect (± SEM) of A5CDQ (3), A6MDQ (6) and A6FDQ (11) at 1.0, 3.0 and 3.0 mg/kg, respectively for jumps and vertical plane (V-plane) entries in the locomotor activity assay	S7
**Figure S-7.** Sequence alignment of hOCT3, mOCT3, hOCT2 and hOCT1	S8
**Table S-1.** Percent identity matrix of hOCT3, mOCT3, hOCT2 and hOCT1	S9
**References**	S9

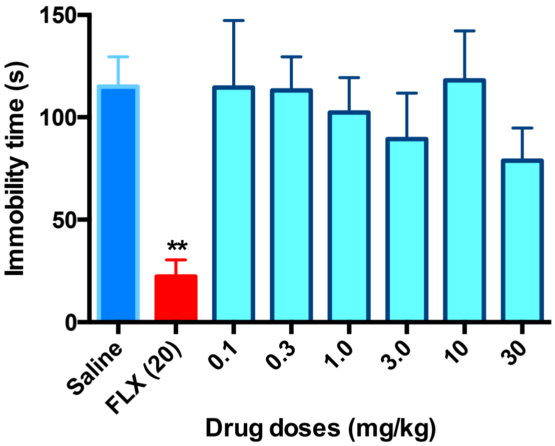

Figure S-1. Effect (± SEM) of A8CDQ (**4**) on duration of immobility in the mouse TST. A8CDQ (**4**) did not significantly reduce the duration of immobility at tested doses (0.1-30 mg/kg) compared to saline according to one-way analysis of variance (ANOVA). For FLX (fluoxetine) ($F_{5,50}$ = 3.714, p = 0.0062), Dunnett's post-hoc test (** *p* < 0.01).

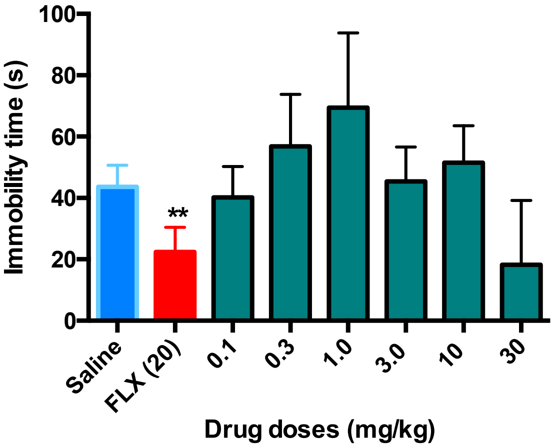

Figure S-2. Effect (± SEM) of ADQ (**5**) on duration of immobility in the mouse TST. ADQ (**5**) did not significantly reduce the duration of immobility at any of the doses tested (0.1-30 mg/kg) compared to saline according to one-way analysis of variance (ANOVA). For FLX (fluoxetine) ($F_{5,50}$ = 3.714, p = 0.0062), Dunnett's post-hoc test (** *p* < 0.01).

Locomotor Activity Assay. Because locomotor stimulants might influence tail suspension time, TruScan control and data acquisition software were employed to record a number of different parameters, out of which the following eleven parameters were analyzed: movement episodes, movement time (s), movement distance (cm), ambulatory velocity (cm/s), margin distance (cm), margin time (s), center distance (cm), center time (s), center entries, jumps and vertical plane (V-plane) entries. The first four parameters listed, movement episodes, movement time (s), movement distance (cm) and ambulatory velocity (cm/s) are altered upon administration of stimulants.[S1,S2] The movement time (s), distance (cm) and ambulatory velocity (cm/s) are increased, whereas the movement episodes reduce in number.[S1,S2] Increases in margin distance (cm) and margin time (s) and a decrease in center distance (cm), center time (s) and center entries are indicative of anxiety or anxiogenic effects.[S1,S3,S4] V-plane entries correspond to rearing behavior and jumps might indicate general responsiveness of mice.[S1,S5,S6] The unpaired two-tailed t-test was used to compare the mean values of the parameters evaluated for saline and the compounds tested (A5CDQ (**3**; 1.0 mg/kg), A6MDQ (**6**; 3.0 mg/kg) and A6FDQ (**11**; 3.0 mg/kg)) to determine if differences observed were statistically significant.

The results indicated an overall lack of locomotor stimulant effect (Figures S-3 – S-6) for all three 2-aminodihydroquinazoline analogs, A5CDQ (**3**), A6MDQ (**6**) and A6FDQ (**11**) at the doses examined.

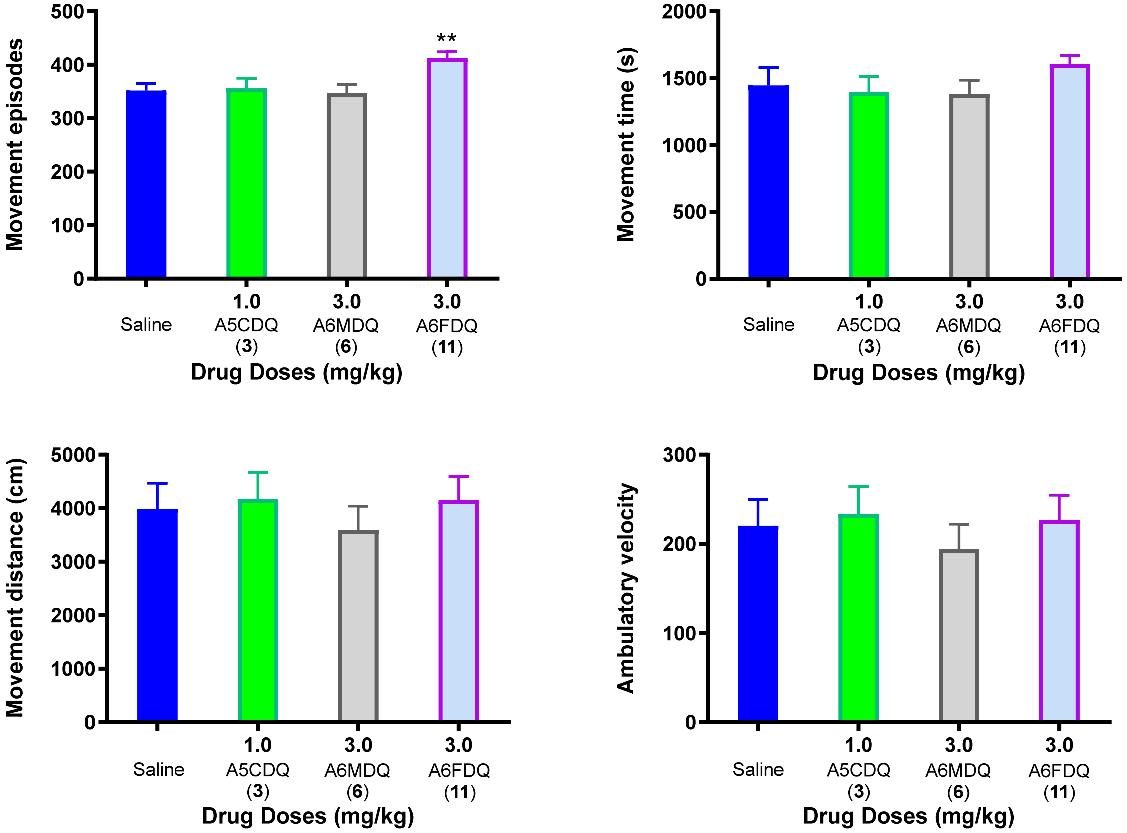

Figure S-3. Effect (± SEM) of A5CDQ (**3**), A6MDQ (**6**) and A6FDQ (**11**) at 1.0, 3.0 and 3.0 mg/kg, respectively for movement episodes, movement time (s), movement distance (cm) and ambulatory velocity (cm/s) in the locomotor activity assay. No significant changes were observed for the parameters for A5CDQ (**3**), A6MDQ (**6**) and A6FDQ (**11**) compared to saline except for movement episodes for A6FDQ (**11**) according to unpaired two-tailed t-test.

Unpaired two-tailed t-test, for movement episodes: A5CDQ (**3**) $t(15) = 0.1749$, $p = 0.8635$, A6MDQ (**6**) $t(15) = 0.2331$, $p = 0.8188$, A6FDQ (**11**) $t(13) = 3.416$, $p = 0.0042$; for movement time (s): A5CDQ (**3**) $t(16) = 0.2698$, $p = 0.7908$, A6MDQ (**6**) $t(16) = 0.3734$, $p = 0.7137$, A6FDQ (**11**) $t(15) = 1.027$, $p = 0.3207$; movement distance (cm): A5CDQ (**3**) $t(15) = 0.2677$, $p = 0.7925$, A6MDQ (**6**) $t(15) = 0.6018$, $p = 0.5563$, A6FDQ (**11**) $t(15) = 0.2628$, $p = 0.7963$; ambulatory velocity (cm/s): A5CDQ (**3**) $t(15) = 0.3027$, $p = 0.7663$, A6MDQ (**6**) $t(15) = 0.6425$, $p = 0.5302$, A6FDQ (**11**) $t(15) = 0.1642$, $p = 0.8718$.

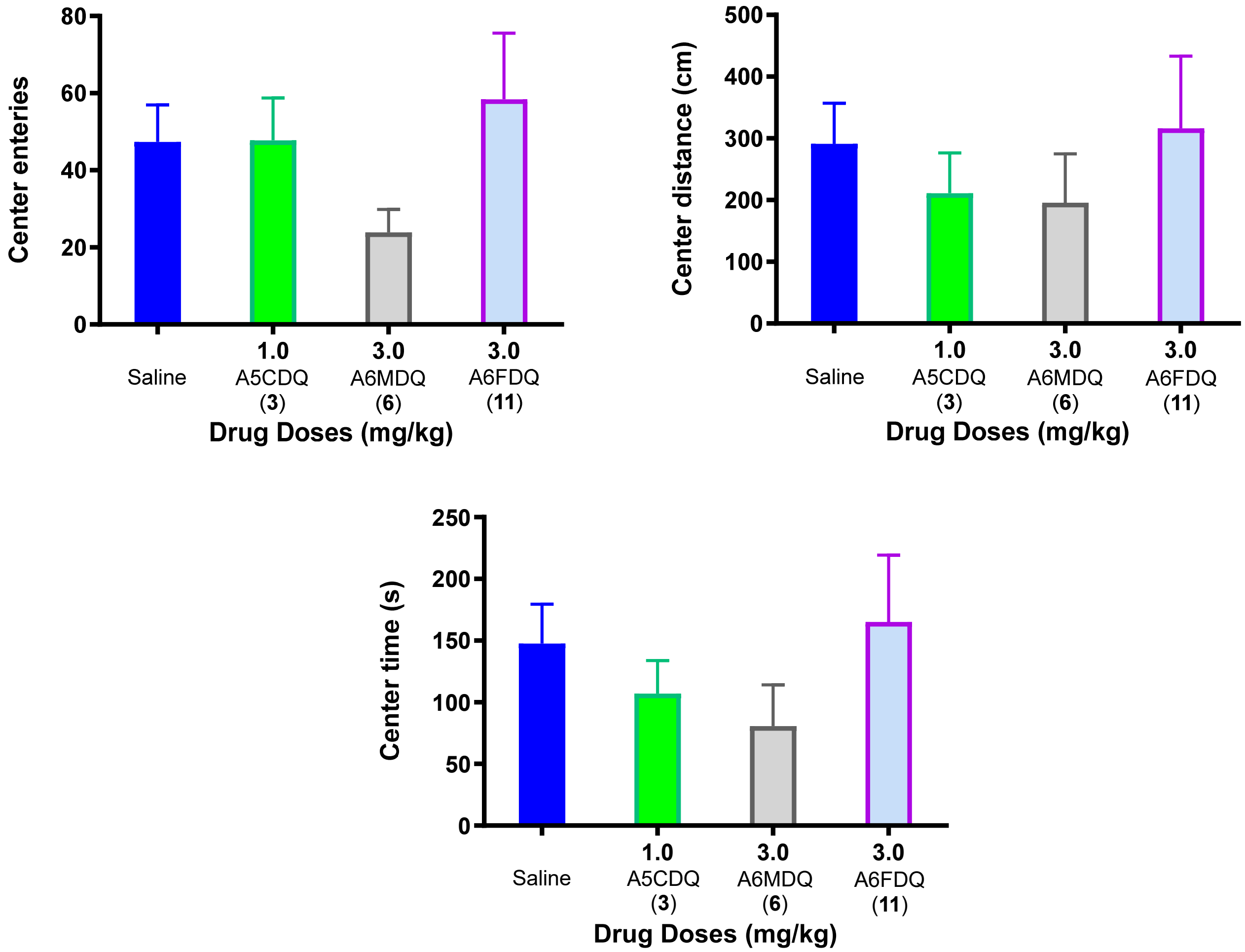

Figure S-4. Effect (± SEM) of A5CDQ (**3**), A6MDQ (**6**) and A6FDQ (**11**) at 1.0, 3.0 and 3.0 mg/kg, respectively for center entries, center distance (cm) and center time (s) in the locomotor activity assay. No significant changes were observed for the parameters for A5CDQ (**3**), A6MDQ (**6**) and A6FDQ (**11**) compared to saline according to unpaired two-tailed t-test.
Unpaired two-tailed t-test, for center entries: A5CDQ (**3**) $t(15) = 0.0272$, $p = 0.9786$, A6MDQ (**6**) $t(14) = 2.078$, $p = 0.0566$, A6FDQ (**11**) $t(15) = 0.5450$, $p = 0.5938$; for center distance (cm): A5CDQ (**3**) $t(16) = 0.8572$, $p = 0.4040$, A6MDQ (**6**) $t(16) = 0.9231$, $p = 0.3697$, A6FDQ (**11** $t(16) = 0.1882$, $p = 0.8531$; center time (s): A5CDQ (**3**) $t(16) = 0.9709$, $p = 0.3460$, A6MDQ (**6**) $t(15) = 1.445$, $p = 0.1690$, A6FDQ (**11**) $t(16) = 0.2768$, $p = 0.7854$.

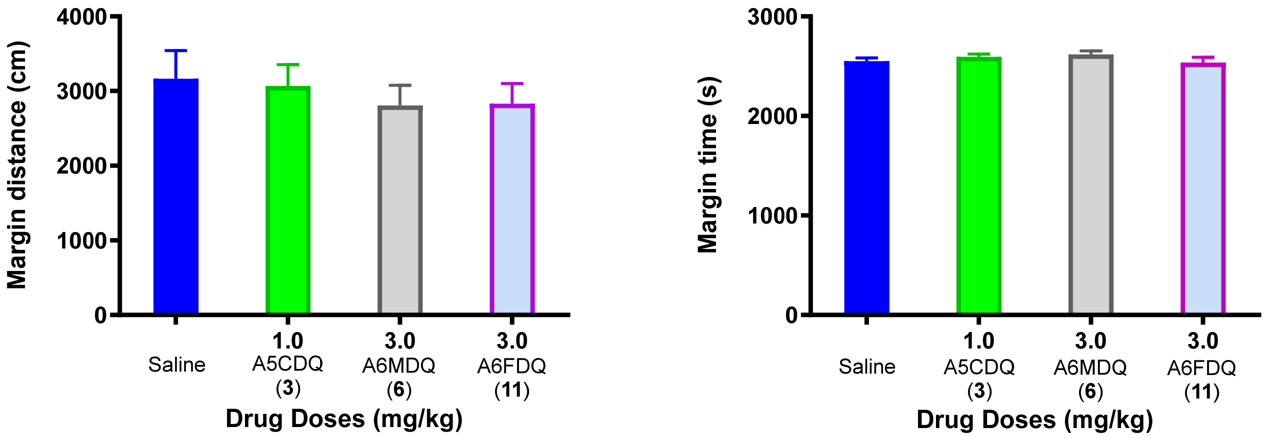

Figure S-5. Effect (± SEM) of A5CDQ (**3**), A6MDQ (**6**) and A6FDQ (**11**) at 1.0, 3.0 and 3.0 mg/kg, respectively for margin distance (cm) and margin time (s). No significant changes were observed for the parameters for A5CDQ (**3**), A6MDQ (**6**) and A6FDQ (**11**) compared to saline according to unpaired two-tailed t-test.

Unpaired two-tailed t-test, for margin distance (cm): A5CDQ (**3**) $t(16) = 0.2085$, $p = 0.8374$, A6MDQ (**6**) $t(16) = 0.7735$, $p = 0.4505$, A6FDQ (**11**) $t(16) = 0.7240$, $p = 0.4795$; for margin time (s): A5CDQ (**3**) $t(16) = 0.9709$, $p = 0.3460$, A6MDQ (**6**) $t(15) = 1.445$, $p = 0.1690$, A6FDQ (**11**) $t(16) = 0.2768$, $p = 0.7854$.

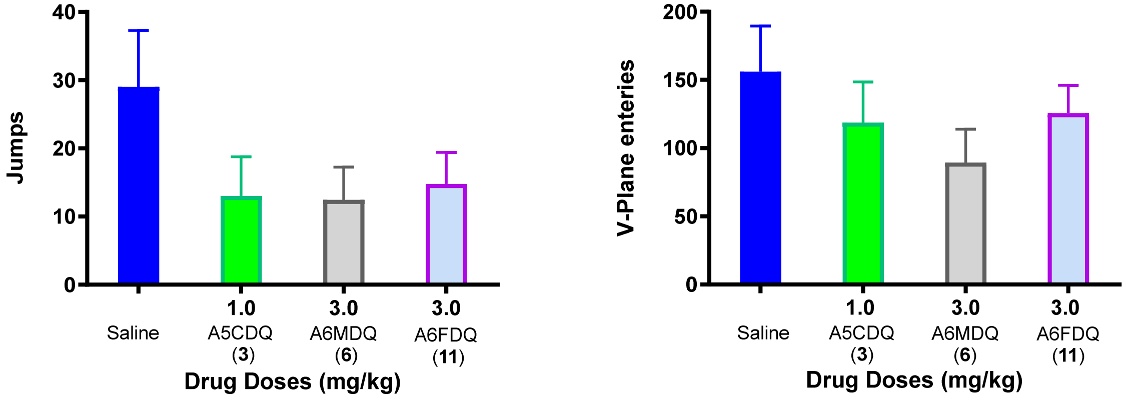

Figure S-6. Effect (± SEM) of A5CDQ (**3**), A6MDQ (**6**) and A6FDQ (**11**) at 1.0, 3.0 and 3.0 mg/kg, respectively for jumps and vertical plane (V-plane) entries in the locomotor activity assay. No significant changes were observed for the parameters for A5CDQ (**3**), A6MDQ (**6**) and A6FDQ (**11**) compared to saline according to unpaired two-tailed t-test.
Unpaired two-tailed t-test, for jumps: A5CDQ (**3**) $t(15) = 1.546$, $p = 0.1429$, A6MDQ (**6**) $t(16) = 1.730$, $p = 0.1029$, A6FDQ (**11**) $t(16) = 1.498$, $p = 0.1536$; for V-Plane entries: A5CDQ (**3**) $t(16) = 0.8320$, $p = 0.4177$, A6MDQ (**6**) $t(16) = 1.607$, $p = 0.1276$, A6FDQ (**11**) $t(16) = 0.7756$, $p = 0.4493$.

```
hOCT3   -MPSFDEALQRVGEFGRFQRRVFLLLCLTGVTFAFLFVGVVFLGTQPDHYWCRGPSAAAL   59
mOCT3   -MPTFDQALRKAGEFGRFQRRVFLLLCLTGVTFAFLFVGVVFLGSQPDYYWCRGPRATAL   59
hOCT2   MPTTVDDVLEHGGEFHFFQKQMFFLLALLSATFAPIYVGIVFLGFTPDH-RCRSPGVAEL   59
hOCT1   -MPTVDDILEQVGESGWFQKQAFLILCLLSAAFAPICVGIVFLGFTPDH-HCQSPGVAEL   58
           :.*: *.: **   **:: *::*.* ..:** : **:**** **:  *:.* .: *

hOCT3   AERCGWSPEEEWNRTAPASRGPEPPERRGRCQRYLLEAANDSASATSALSCADPLAAFPN   119
mOCT3   AERCAWSPEEEWNLTTPELHVPAERRGQGHCHRYLLEATNTSSE-----LSCDPLTAFPN   114
hOCT2   SLRCGWSPAEELNYTVPGP-GPAGEASPRQCRRYEVDWN------QSTFDCVDPLASLDT   112
hOCT1   SQRCGWSPAEELNYTVPGL-GPAGEAFLGQCRRYEVDWN------QSALSCVDPLASLAT   111
        : **.*** ** * *.*    *       :*:** ::              . ***::: .

hOCT3   --RSAPLVPCRGGWRYAQAHSTIVSEFDLVCVNAWMLDLTQAILNLGFLTGAFTLGYAAD   177
mOCT3   --RSAPLVSCSGDWRYVETHSTIVSQFDLVCSNAWMLDLTQAILNLGFLAGAFTLGYAAD   172
hOCT2   NRSRLPLGPCRDGWVYETPGSSIVTEFNLVCANSWMLDLFQSSVNVGFFIGSMSIGYIAD   172
hOCT1   NRSHLPLGPCQDGWVYDTPGSSIVTEFNLVCADSWKLDLFQSCLNAGFLFGSLGVGYFAD   171
             **  * ..* *    *:**::*:*** ::* *** *: :* **: *:: :** **

hOCT3   RYGRIVIYLLSCLGVGVTGVVVAFAPNFPVFVIFRFLQGVFGKGTWMTCYVIVTEIVGSK   237
mOCT3   RYGRLIIYLISCFGVGITGVVVAFAPNFSVFVIFRFLQGVFGKGAWMTCFVIVTEIVGSK   232
hOCT2   RFGRKLCLLTTVLINAAAGVLMAISPTYTWMLIFRLIQGLVSKAGWLIGYILITEFVGRR   232
hOCT1   RFGRKLCLLGTVLVNAVSGVLMAFSPNYMSMLLFRLLQGLVSKGNWMAGYTLITEFVGSG   231
        *:** :  * : :  . :**::*::*.:  :::**::**:..*. *:  : ::**:**

hOCT3   QRRIVGIVIQMFFTLGIIILPGIAYFIPNWQGIQLAITLPSFLFLLYYWVVPESPRWLIT   297
mOCT3   QRRIVGIVIQMFFTLGIIILPGIAYFTPSWQGIQLAISLPSFLFLLYYWVVPESPRWLIT   292
hOCT2   YRRTVGIFYQVAYTVGLLVLAGVAYALPHWRWLQFTVSLPNFFFLLYYWCIPESPRWLIS   292
hOCT1   SRRTVAIMYQMAFTVGLVALTGLAYALPHWRWLQLAVSLPTFLFLLYYWCVPESPRWLLS   291
         ** *.*. *: :*:*:: * *:** * *: :*::::**.*:****** :*******::

hOCT3   RKKGDKALQILRRIAKCNGKYLSSNYSEITVTD--EEVSNPSFLDLVRTPQMRKCTLILM   355
mOCT3   RKQGEKALQILRRVAKCNGKHLSSNYSEITVTD--EEVSNPSCLDLVRTPQMRKCTLILM   350
hOCT2   QNKNAEAMRIIKHIAKKNGKSLPASLQRLRLEEETGKKLNPSFLDLVRTPQIRKHTMILM   352
hOCT1   QKRNTEAIKIMDHIAQKNGKLPPADLKMLSLEEDVTEKLSPSFADLFRTPRLRKRTFILM   351
        :::. :*::*: ::*: ***   :. . : : :   :  .**  **.***::** *:***

hOCT3   FAWFTSAVVYQGLVMRLGIIGGNLYIDFFISGVVELPGALLILLTIERLGRRLPFAASNI   415
mOCT3   FAWFTSAVVYQGLVMRLGLIGGNLYIDFFISGLVELPGALLILLTIERLGRRLPFAASNI   410
hOCT2   YNWFTSSVLYQGLIMHMGLAGDNIYLDFFYSALVEFPAAFMIILTIDRIGRRYPWAASNM   412
hOCT1   YLWFTDSVLYQGLILHMGATSGNLYLDFLYSALVEIPGAFIALITIDRVGRIYPMAMSNL   411
        : ***.:*:****::::*  ..*:*:**: *.:**:*.*:: ::**:*:**  * * **:

hOCT3   VAGVACLVTAFLPEAGIAWLRTTVATLGRLGITMAFEIVYLVNSELYPTTLRNFGVSLCS   475
mOCT3   VAGVSCLVTAFLPE-GIPWLRTTVATLGRLGITMAFEIVYLVNSELYPTTLRNFGVSLCS   469
hOCT2   VAGAACLASVFIPG-DLQWLKIIISCLGRMGITMAYEIVCLVNAELYPTFIRNLGVHICS   471
hOCT1   LAGAACLVMIFISP-DLHWLNIIIMCVGRMGITIAIQMICLVNAELYPTFVRNLGVMVCS   470
        :**.:**.  *:    .: **.  :  :**:***:* ::: ***:***** :**:** :**

hOCT3   GLCDFGGIIAPFLLFRLAAVWLELPLIIFGILASICGGLVMLLPETKGIALPETVDDVEK   535
mOCT3   GLCDFGGIIAPFLLFRLAAIWLELPLIIFGILASVCGGLVMLLPETKGIALPETVEDVEK   529
hOCT2   SMCDIGGIITPFLVYRLTNIWLELPLMVFGVLGLVAGGLVLLLPETKGKALPETIEEAEN   531
hOCT1   SLCDIGGIITPFIVFRLREVWQALPLILFAVLGLLAAGVTLLLPETKGVALPETMKDAEN   530
        .:**:****:**::**  :*  ***::*.:*. :..*:.:******* *****:.:.*:

hOCT3   LGSPHSCKCGRNKKTPVSRSHL---   557
mOCT3   LGSSQLHQCGRKKKTQVSTSDV---   551
hOCT2   MQRPRKNKEKMI-YLQVQKLDIPLN   555
hOCT1   LGRKAKPKENTI-YLKVQTSEPSGT   554
        :      :        *.  .
```

Figure S-7. Sequence alignment of hOCT3, mOCT3, hOCT2 and hOCT1.

Table S-1. Percent identity matrix of hOCT3, mOCT3, hOCT2 and hOCT1.

	**hOCT3**	**mOCT3**	**hOCT2**	**hOCT1**
**hOCT3**	100.00	86.57	49.73	49.73
**mOCT3**	86.57	100.00	48.43	47.15
**hOCT2**	49.73	48.43	100.00	69.68
**hOCT1**	49.73	47.15	69.68	100.00

References

S1. Young, R., Glennon, R. A. (2008) MDMA (N-methyl-3,4-methylenedioxyamphetamine) and its stereoisomers: Similarities and differences in behavioral effects in an automated activity apparatus in mice. *Pharmacol. Biochem. Behav. 88*, 318–331.

S2. Berlyne, D. E. *Conflict, Arousal, and Curiosity*. McGraw-Hill: New York, 1960.

S3. Simon, P., Dupuis, R., Costentin, J. (1994) Thigmotaxis as an index of anxiety in mice. Influence of dopaminergic transmissions. *Behav. Brain Res. 61*, 59–64.

S4. Treit, D., Fundytus, M. (1988) Thigmotaxis as a test for anxiolytic activity in rats. *Pharmacol. Biochem. Behav. 31*, 959–962.

S5. Walsh, R.N., Cummins, R.A. (1976) The open-field test: A critical review. *Psychol. Bull. 83*, 482–504.

S6. Lát, J., Gollová-Hémon, E. (1969) Permanent effects of nutritional and endocrinological intervention in early ontogeny on the level of nonspecific excitability and on lability (emotionality). *Ann. N. Y. Acad. Sci. 159*, 710–720.
